# Overcoming challenges in cartilage regeneration: The role of chondrogenic inducers

**DOI:** 10.1002/btm2.70079

**Published:** 2025-09-29

**Authors:** Manh Tuong Nguyen, Stan Gronthos, Yunpeng Zhao, Vashe Chandrakanthan, Vi Khanh Truong, Krasimir Vasilev

**Affiliations:** ^1^ Biomedical Nanoengineering Laboratory, College of Medicine and Public Health Flinders University Adelaide South Australia Australia; ^2^ School of Biomedicine, Faculty of Health and Medical Sciences The University of Adelaide Adelaide South Australia Australia; ^3^ Precision Medicine Theme South Australian Health and Medical Research Institute Adelaide South Australia Australia; ^4^ Department of Orthopedics Qilu Hospital of Shandong University Shandong People's Republic of China; ^5^ Adelaide Medical School, Faculty of Health and Medical Sciences The University of Adelaide Adelaide South Australia Australia; ^6^ School of Medical Sciences University of New South Wales Sydney New South Wales Australia; ^7^ Precision Medicine South Australian Health and Medical Research Institute Adelaide South Australia Australia; ^8^ Healthcare Engineering Innovation Group, Department of Biomedical Engineering and Biotechnology Khalifa University Abu Dhabi UAE

**Keywords:** cartilage repair, chondrogenesis, chondrogenic inducers, growth factors, stem cells

## Abstract

Cartilage regeneration presents unique challenges due to its avascular structure, sparse cell population, and limited regenerative capacity. Recent years have seen significant advancements in the field, which warrant an integrated review that connects chondrogenesis and its practical application. This review aims to deliver comprehensive and analytical guidelines for understanding the complex process of chondrogenesis, emphasizing its critical role in cartilage regeneration. It reviews key inducers such as growth factors, mechanical stimuli, hypoxia, and electric fields, as well as their synergistic integration with biomaterials to facilitate effective strategies for repairing and regenerating damaged cartilage tissue. In addition to exploring these advancements, the paper also provides a critical evaluation of current methods used to assess chondrogenesis in in vitro and in vivo models, identifying gaps and possibilities for improvement. A particular focus is placed on addressing the translational challenges that hinder the clinical implementation of cutting‐edge research findings, offering actionable strategies to bridge the gap between laboratory discoveries and patient outcomes. By examining emerging trends and consolidating recent innovations, this review aims to offer a holistic perspective on cartilage repair. It serves as a guide for researchers and clinicians, advocating for collaborative, interdisciplinary approaches to advance the field and deliver improved therapeutic solutions for cartilage‐related conditions.

**Translational Impact Statements**

Understanding the challenges of cartilage regeneration and the role of chondrogenesis inducers in cartilage tissue repair is crucial for developing an effective biomaterial‐based strategy, ensuring its successful translation into clinical practice.

## 1 INTRODUCTION

Cartilage injuries or diseases significantly affect human health worldwide.^1^ Musculoskeletal disorders, which include conditions that affect joints, bones, and muscles, affect over 1.7 billion individuals worldwide.^2^ In the United States, knee cartilage injuries impact around 900,000 individuals each year, leading to over 200,000 surgical procedures.^3^ The biggest challenge in cartilage regeneration following loss or damage lies in its unique histology: the tissue's limited regenerative capability. Unlike other tissues, cartilage has a low cell density, a large extracellular matrix (ECM), and lacks both blood vessels and neuronal cells, making effective regeneration particularly difficult.^4^

Although numerous therapeutic approaches have been developed, no existing pharmacological or surgical solutions for cartilage repair are capable of fully restoring damaged articular cartilage to its natural structure and functionality.^5^ Consequently, there is a critical need for external interventions to repair and restore native tissue functionality. Chondrogenesis, the process where mesenchymal stem cells (MSCs) differentiate into chondrocytes, represents a promising approach. Inducers of chondrogenesis could potentially facilitate the differentiation of stem cells and progenitor cells into chondrocytes at injury sites, thereby offering a viable treatment pathway for cartilage injuries or diseases. Several studies have demonstrated the potential of chondrogenic induction in promoting cartilage repair. In 1959, Pridie introduced subchondral drilling as a marrow‐stimulating technique, which has since been utilized to treat cartilage defects in patients with osteoarthritis and focal chondral lesions.^6^ This method is considered one of the simplest ways to stimulate chondrogenesis. Over the last decade, significant advancements have been made in developing chondrogenesis inducers, which are fundamental for cartilage generation and repair.

Among the earliest discoveries, proteins from the transforming growth factor beta (TGF‐β) family play crucial roles in regulating the chondrogenesis process across various stages, including condensation, proliferation, maturation, and the preservation of articular chondrocytes.^7^ Exploration into mechanically induced chondrogenesis began taking shape in 2011, when a study demonstrated that shear force can significantly increase chondrogenic gene expression via dynamic compression in both in vitro and in vivo.^8^ In 2012, Adetola and his colleagues found that hypoxia‐mediated conditions could enhance the chondrogenic potential of MSCs.^9^ Concurrently, Johnson et al. contributed to the chondrogenesis inducers library by identifying Kartogenin (KGN) after screening 22,000 drug‐like molecules.^10^ In 2016, a low‐frequency electric field was found to be a reliable and economical inducer in the cartilage differentiation of human adipose‐derived stem cells.^11^ Up to now, the concept of chondrogenesis inducers has evolved toward the use of combination biomaterials that enhance chondrogenesis to promote cartilage repair.^12^, ^13^

Currently, while over 3000 review papers indexed in Scopus focus on “cartilage regeneration,” there appears to be a lack of a comprehensive, integrative analysis that spans the entire process from chondrogenesis to clinical application.^14^, ^15^, ^16^ Existing reviews largely neglect a critical evaluation and comparison of chondrogenesis assessment techniques across both in vitro and in vivo models, particularly in relation to the intricate nature of human tissue.^14^, ^16^, ^17^ This oversight represents a substantial knowledge gap, especially in the context of the translational challenges that hinder the application of research findings to clinical practice.

This review explores the fundamental and applied aspects of cartilage regeneration and chondrogenesis by analyzing the latest advancements in the field, focusing on key inducers such as growth factors, small molecules, and mechanical stimuli, and examining their interplay with biomaterials to enhance cartilage repair. The review critically examines current methodologies for assessing chondrogenesis in both in vitro and in vivo settings, identifying limitations and probable areas for refinement. Particular emphasis is given to the translational challenges that impede the clinical application of research findings, offering insights into strategies for overcoming these barriers. By consolidating the latest developments and providing a comprehensive analysis of emerging trends, this review aims to serve as a valuable reference for researchers and clinicians. It highlights the promise of innovative approaches to cartilage regeneration and underscores the importance of interdisciplinary efforts in advancing the field toward improved clinical outcomes.

## 2 UNDERSTANDING CARTILAGE TISSUE AND BARRIERS TO EFFECTIVE REPAIR

**FIGURE 1:**
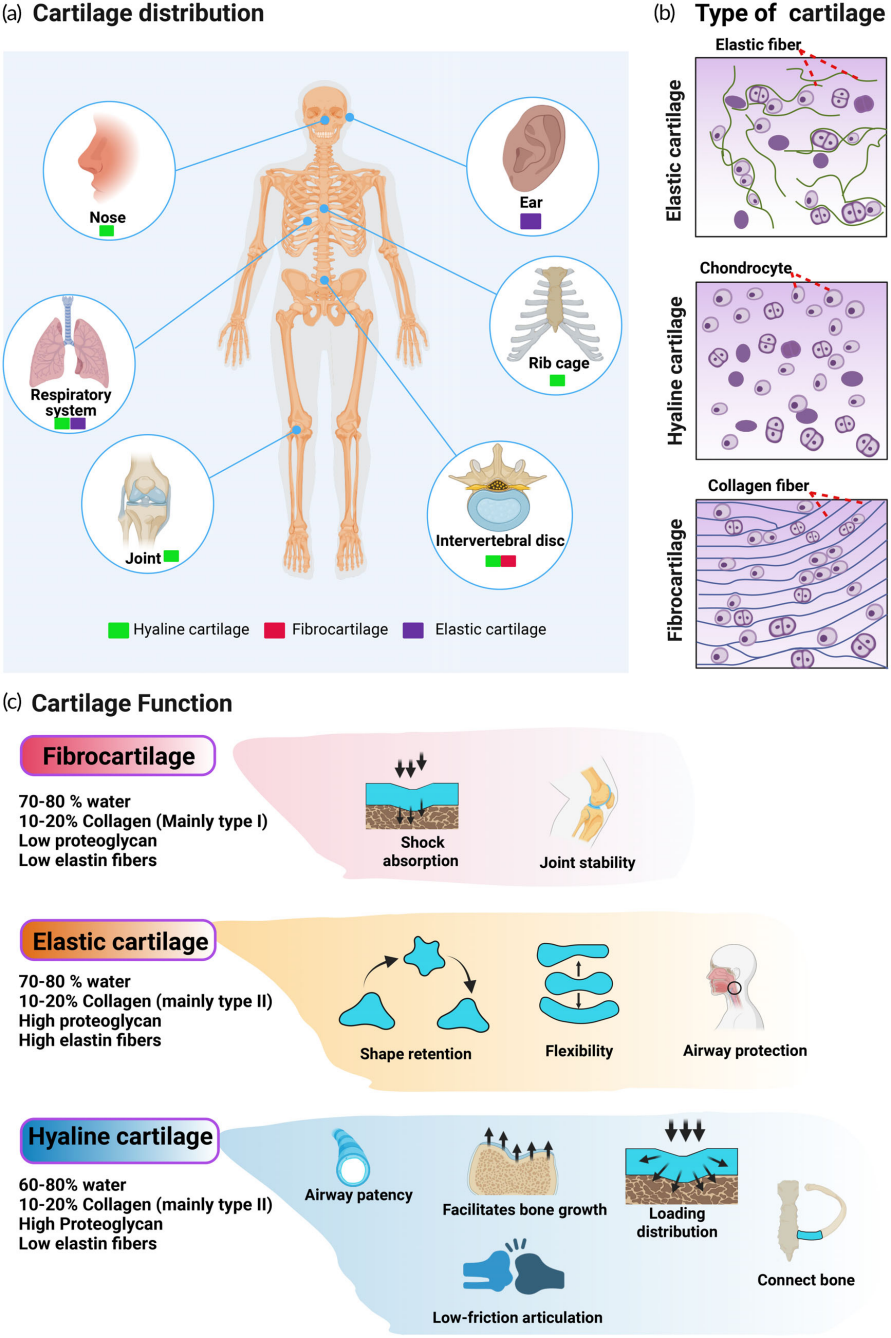
Cartilage types: anatomical distribution, microscopic features, and functional composition: This figure illustrates key distinctions among hyaline cartilage, fibrocartilage, and elastic cartilage. (a) Maps their anatomical localization: hyaline cartilage (green) is found in joints, the respiratory tract, nasal structures, and costal regions; fibrocartilage (red) is in intervertebral discs and select joint areas; and elastic cartilage (purple) resides in the ear and epiglottis. (b) Presents representative histological images, highlighting dense elastic fiber networks in elastic cartilage, clustered chondrocytes in a smooth matrix for hyaline cartilage, and parallel collagen bundles interspersed with chondrocytes in fibrocartilage. (c) Summarizes their biochemical composition and functional roles: fibrocartilage features high type I collagen and low proteoglycan content, supporting shock absorption and joint stability; elastic cartilage contains abundant elastin and proteoglycan for structural flexibility; and hyaline cartilage is rich in type II collagen and proteoglycan, contributing to joint articulation, load distribution, and airway patency. This figure was constructed using BioRender.

Cartilage is a specialized connective tissue primarily characterized by its durability and flexibility. It is found in anatomical regions including joints, costal areas, external ears, nasal structures, the respiratory tract, and other locations throughout the body (Figure 1a). Cartilage is distributed in three types—hyaline cartilage, elastic cartilage, and fibrocartilage. Although each type possesses unique structural and functional properties, they can coexist or be found in adjacent regions depending on biomechanical requirements. For instance, hyaline and fibrocartilage may both contribute to joint architecture (intervertebral disc), while elastic cartilage can be located near hyaline structures within the respiratory tract (Figure 1a). Structurally, all three types contain chondrocytes housed within lacunae, embedded in a dense ECM. However, their matrix composition varies significantly; that is, elastic cartilage contains abundant elastin fibers, hyaline cartilage features a homogeneous ECM, while fibrocartilage is characterized by densely packed type I collagen fibers (Figure 1b).^18^ As illustrated in Figure 1c, fibrocartilage, elastic cartilage, and hyaline cartilage exhibit distinct biomechanical roles stemming from their unique matrix compositions. Fibrocartilage, with high collagen type I and low proteoglycan and elastin content, provides tensile strength and shock absorption in stress‐bearing regions like intervertebral discs.^19^ Elastic cartilage, rich in elastin and proteoglycans, enables flexibility and shape retention in structures like the ear and epiglottis. Hyaline cartilage, defined by high proteoglycan and moderate collagen type II levels, facilitates smooth articulation and load distribution in joints and respiratory structures.^20^, ^21^

**FIGURE 2:**
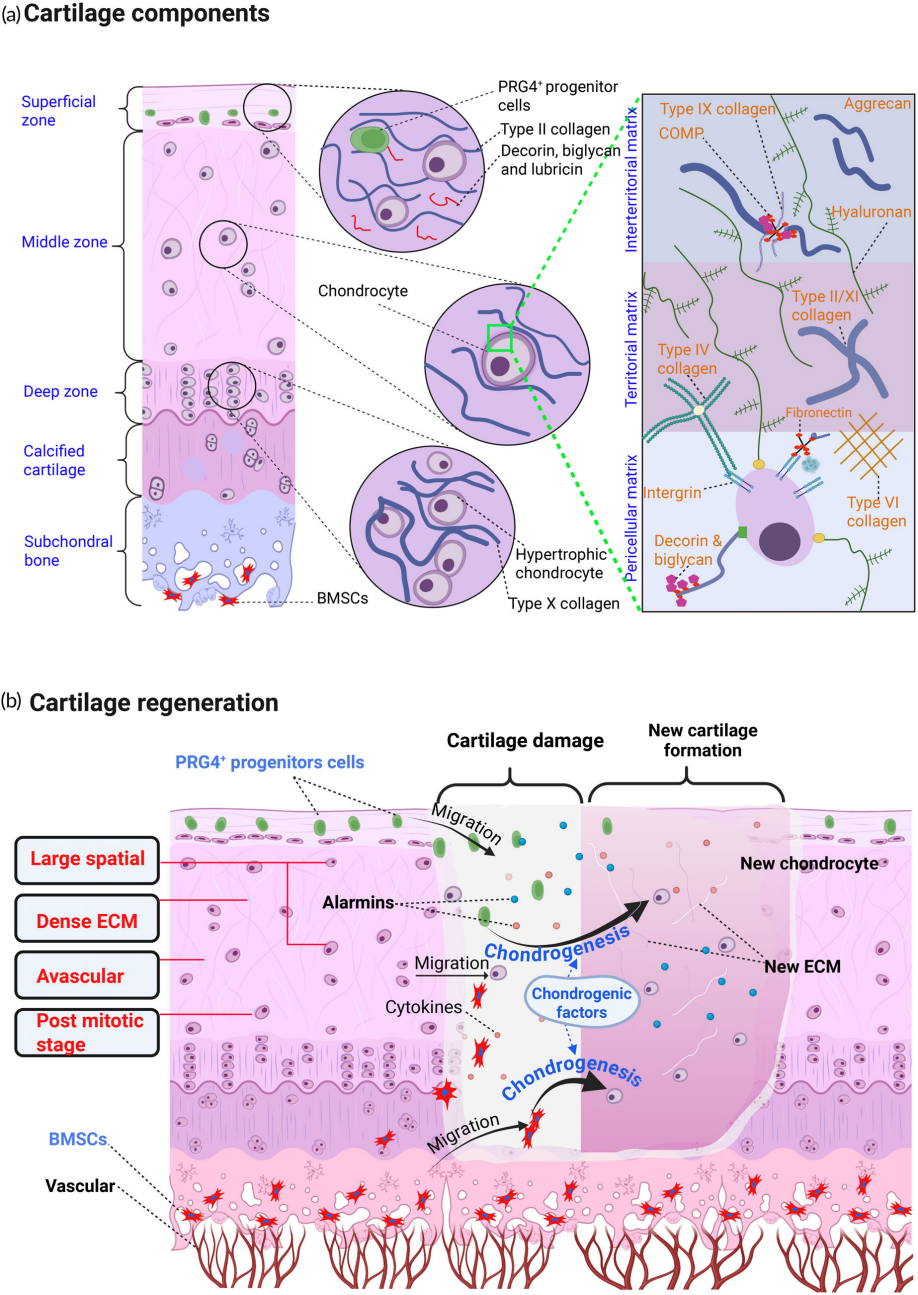
Zonal organization of cartilage and key mechanisms in cartilage regeneration. This figure illustrates the structural complexity of cartilage and its dynamic regenerative mechanisms. (a) Depicts the zonal architecture of cartilage tissue, highlighting the superficial, middle, deep, calcified, and subchondral regions. Each zone contains distinct cellular and matrix components: the superficial zone harbors PRG4^+^ progenitor cells, lubricin, type II collagen, decorin, and biglycan; the middle zone contains mature chondrocytes; the deep zone includes hypertrophic chondrocytes and type X collagen; the calcified cartilage and subchondral bone host vascular structures and bone marrow‐derived stromal cells (BMSCs). An inset further details the pericellular, territorial, and interterritorial matrices, showing associated proteins such as type IX/XI collagen, COMP, aggrecan, fibronectin, integrins, and type VI collagen. (b) Outlines key regenerative processes initiated by PRG4^+^ progenitor cells and BMSCs, including migration, matrix synthesis, alarmin, and cytokine signaling, and chondrogenic differentiation leading to new cartilage formation. Despite these mechanisms, cartilage regeneration remains constrained by tissue‐specific factors such as large spatial heterogeneity, dense extracellular matrix composition, avascularity, and the post‐mitotic nature of resident chondrocytes. This figure was constructed using BioRender. ECM, extracellular matrix.

As illustrated in Figure 2a, cartilage exhibits a hierarchical structural organization that reflects region‐specific functional specialization. The superficial layer contains PRG4^+^ progenitor cells and lubricating macromolecules such as lubricin, decorin, and biglycan, which together maintain surface integrity and enable low‐friction articulation.^22^, ^23^ Deeper within the tissue, the intermediate region features mature chondrocytes embedded in a matrix dominated by type II collagen, which provides resistance to compressive forces and supports ECM turnover.^24^ The basal layer, enriched with hypertrophic chondrocytes and type X collagen, signals the onset of mineralization and structural transition toward bone. Immediately beneath this, the calcified cartilage and subchondral bone house bone marrow stromal cells (BMSCs) involved in mechanical load distribution and anchorage. Throughout these structural layers, the ECM composition varies dynamically, comprising aggrecan (ACAN), COMP, fibronectin, and several collagen types (II, IV, VI, IX, and X), which together contribute to the mechanical resilience, lubrication, and osseous integration capabilities of the tissue, ensuring the maintenance of cartilage homeostasis.^24^

Despite its mechanically adaptive and structurally complex architecture, articular cartilage displays a profoundly limited intrinsic regenerative capacity. This is primarily attributable to its avascularity, the low cellular density of its ECM‐rich environment, and the terminally differentiated, post‐mitotic phenotype of resident chondrocytes (Figure 2b).^19^ In addition, cartilage regeneration is further impeded by the dynamic biomechanical environment of articulating joints. Continuous loading, shear forces, and joint movement disrupt the retention of reparative cells and bioscaffold materials at the injury site. Moreover, the surrounding synovial fluid lacks the coagulative factors necessary for fibrin clot formation, which under typical circumstances provides a provisional matrix for cell infiltration and wound stabilization.^25^ Small solutes, including nutrients like glucose and oxygen, can only diffuse through the synovial fluid to reach the chondrocytes.^26^ The compressive forces frequently applied to cartilage enhance the diffusion of nutrients. This indirect method of nutrient delivery significantly contributes to the slow turnover of the ECM, as well as the limited self‐repair ability and slower healing of cartilage compared to other tissues. As a result, spontaneous repair is limited, and therapeutic interventions must overcome these physical and biochemical barriers to restore cartilage integrity.

## 3 THE ROLE OF CHONDROGENESIS IN CARTILAGE REPAIR

Chondrogenesis, or cartilage differentiation, is an essential process for the formation and repair of cartilage tissue. This intricate biological process involves a series of well‐coordinated stages that guide MSCs to differentiate into chondrocytes, the specialized cells responsible for producing cartilage.^27^ This process is fundamental not only during embryonic development but also for successful cartilage regeneration, serving as a vital connection in overcoming the challenges of cartilage repair.

### 3.1 Chondrogenesis in cartilage regeneration

Cartilage regeneration is a coordinated biological process in which MSCs play a central regenerative role by undergoing chondrogenesis to restore tissue integrity (Figure 2b). Injury triggers the release of alarmins and inflammatory mediators, which stimulate chemotactic signals such as stromal cell‐derived factor 1 (SDF‐1), C‐C motif chemokine ligand 2 (CCL2), and C‐X‐C motif chemokine ligand 12 (CXCL12).^28^ These chemotactic cues recruit BMSCs from the subchondral region, as well as PRG4^+^ progenitor cells from the synovial niche or cartilage surface, guiding them toward the injury site where they participate in chondrogenesis by differentiating into chondrocyte‐like cells.^29^ The newly differentiated cells actively synthesize cartilage‐specific ECM components such as type II collagen, ACAN, and COMP, thereby contributing to the structural restoration and functional recovery of the damaged tissue. This process is similar to the development of cartilage but has specific adaptations depending on the regeneration conditions.^30^ In addition, chondrogenesis is the fundamental process that leads to the formation of cartilage during embryonic development.^30^

### 3.2 Chondrogenic differentiation process

**FIGURE 3:**
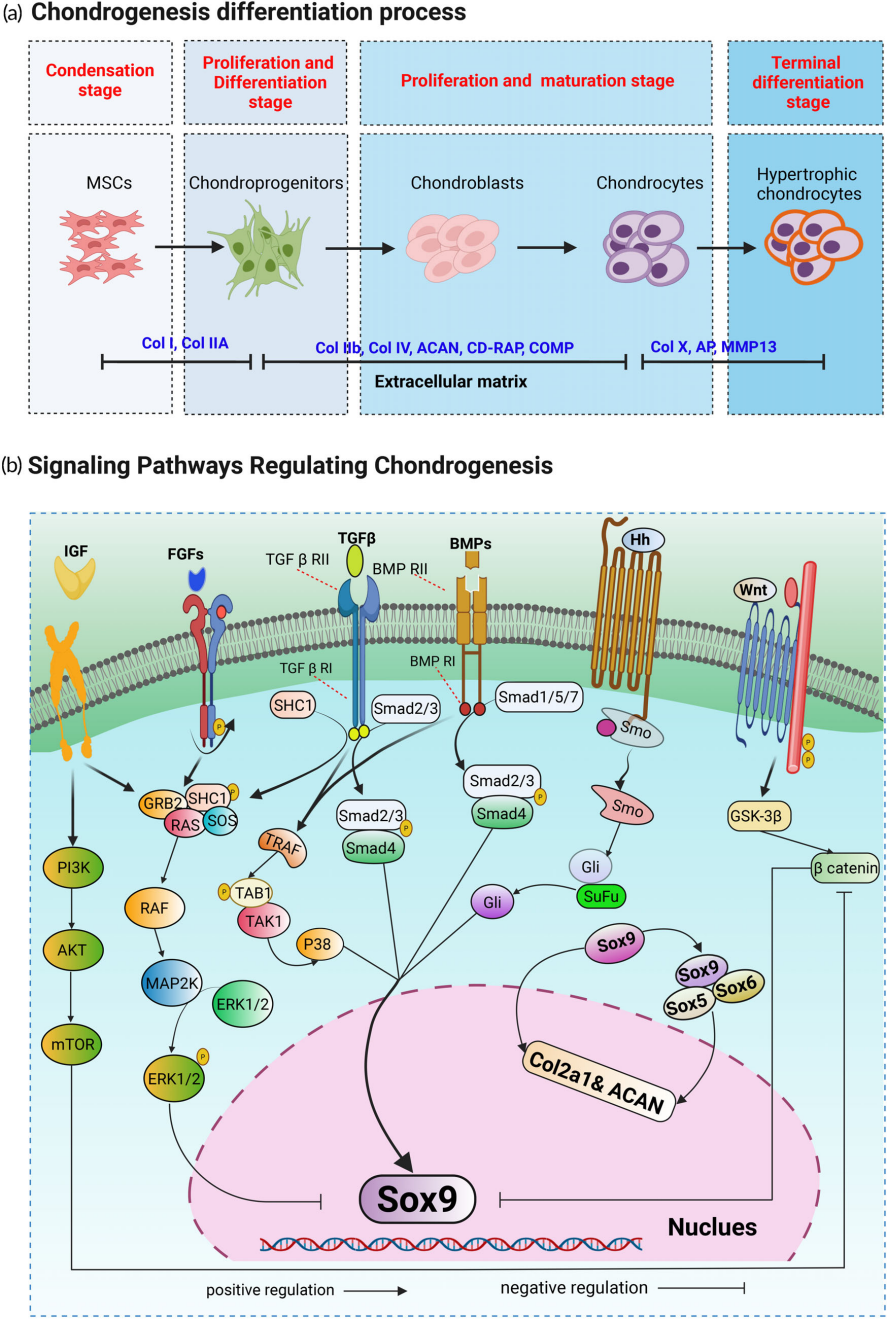
Cellular progression and molecular signaling in chondrogenesis. This figure depicts the cellular and molecular mechanisms governing chondrogenesis, a central process in cartilage formation and repair. (a) Illustrates the sequential differentiation of mesenchymal stem cells (MSCs) into hypertrophic chondrocytes, progressing through condensation, chondroprogenitor expansion, maturation into chondroblasts and chondrocytes, and terminal hypertrophy. Distinct extracellular matrix proteins—including collagen types I, IIA, II, IV, and X, aggrecan (ACAN), cartilage‐derived retinoic acid‐sensitive protein (CD‐RAP), cartilage oligomeric matrix protein (COMP), alkaline phosphatase, and matrix metalloproteinase‐13 (MMP13)—define each stage and support functional transitions. (b) Summarizes key signaling pathways regulating chondrogenesis, including insulin‐like growth factor (IGF), fibroblast growth factors (FGF), transforming growth factor beta (TGF‐β), bone morphogenetic proteins (BMP), Hedgehog, and Wnt. These networks activate intracellular mediators such as phosphoinositide 3‐kinase (PI3K), a family of serine/threonine kinases (Akt), extracellular signal regulated kinases 1 and 2 (ERK1/2), Smad proteins, Gli, and β‐catenin, converging on Sox9 to drive chondrogenic differentiation (e.g., Col2a1 and ACAN). This figure was constructed using BioRender. AP, Activating Enhancer Binding Proteins.

Chondrogenesis progresses through sequential stages, including mesenchymal condensation, cellular differentiation, maturation, and terminal differentiation. As shown in Figure 3a, cartilage development proceeds through a series of tightly regulated stages, from initial cell condensation to terminal hypertrophy. Each stage contributes to shaping the tissue's structural and functional properties. The progression from condensation to early proliferation and differentiation is marked by increased expression of key ECM components, including type I collagen, type II collagen (Col2A1), and ACAN. These matrix proteins play important roles in organizing the cellular environment, supporting early tissue architecture, and providing the mechanical strength necessary for load‐bearing function. Chondrogenesis initiates as mesenchymal cells condense and aggregate, expressing cell adhesion markers such as neural cadherin (N‐cadherin) and neural cell adhesion molecule (NCAM). In addition, master regulatory transcription factors such as SRY (sex‐determining region Y)‐box 9 (Sox9) are upregulated early and regulate collagen II, an important cartilage marker. Cell cycle and proliferation genes are downregulated as condensation progresses to induce cell cycle exit. In the embryonic development of cartilage, mesenchymal progenitor cells initially display a spread morphology. However, after a few days, they begin to adopt a rounded shape and begin forming aggregates.^31^

After condensation, MSCs undergo differentiation into chondrocytes, a process controlled by several growth factors, notably the TGF‐β superfamily and their downstream regulators, the Smads.^32^ Sox9 and Col2A1 continue upregulating during chondrocyte differentiation. In addition, a large proteoglycan (ACAN) is another essential component of the ECM and is upregulated.^33^ At this stage of chondrogenesis, cells continue to reduce spreading and transition to a rounded phenotype. This shape change is facilitated by the surrounding mechanical and chemical environment. In pellet cultures, which provide a three‐dimensional (3D) environment, cells are forced into a compacted shape, reducing cell spreading.^34^

Next, chondrocytes start secreting molecules that form the ECM, which provides both structural stability and biochemical support to the surrounding cells. Key components of the ECM include collagen (mainly type II collagen), ACAN, and glycosaminoglycans.^32^ In vitro, the ECM deposition stage can be observed after 21 days of differentiation.

As chondrogenesis progresses, chondrocytes mature and undergo hypertrophy, increasing in size. This process is regulated by various signaling molecules, including the Indian hedgehog (Ihh), which stimulates chondrocyte proliferation and regulates chondrocyte maturation. Gremlin, which identifies chondro‐ and osteoprogenitor cells and affects the development of arthritis, plays a crucial role during this period by modulating bone morphogenetic proteins (BMP) signaling to maintain proper chondrocyte differentiation and prevent premature hypertrophy.^35^, ^36^ Collagen type X (Col10A1) is a crucial marker for this stage, which is not expressed in healthy cartilage.^37^ Additionally, Runt‐related transcription factor 2 (RUNX2) is a key transcription factor involved in chondrocyte hypertrophy and is upregulated during this stage.^37^ Parathyroid hormone‐related protein (PTHrP) also takes part in the regulation of chondrocyte differentiation and maturation.^38^

The final stage of chondrogenesis involves the apoptosis of hypertrophic chondrocytes and calcification of the cartilage matrix. This stage prepares for the replacement of cartilage with bone during the process of endochondral ossification.^32^

Several keys signaling pathways regulate the chondrogenic differentiation of MSCs, many of which converge on Sox9, a core transcription factor essential for chondrocyte lineage commitment, as shown in Figure 3b. The TGF‐β and BMP families initiate chondrogenesis through Smad‐mediated signaling, driving the expression of cartilage‐specific genes such as Col2a1 and ACAN.^39^ Hedgehog (Hh) signaling, particularly via Ihh and Shh, supports early mesenchymal condensation and reinforces Sox9 activity through GLI transcription factors, often working in tandem with BMP pathways.^40^

Wingless‐related integration site (Wnt) signaling plays a dual role in this process: the canonical β‐catenin pathway tends to inhibit Sox9 expression, while non‐canonical branches, operating through mitogen‐activated protein kinase (MAPK) signaling, can enhance chondrogenic differentiation. Fibroblast growth factors (FGFs) also modulate MSC behavior in a context‐dependent manner—promoting early commitment through fibroblast growth factor receptor (FGFR) activation and downstream phosphoinositide 3‐kinase (PI3K)/Akt signaling, aided by adaptor proteins like FRS2 and Gab1 that support cell survival and ECM production. However, under certain conditions, FGFs can also inhibit chondrogenesis via p38 MAPK signaling. Insulin‐like growth factor (IGF) signaling contributes to matrix synthesis and differentiation through the PI3K/Akt/mTOR pathway, reinforcing the chondrogenic program. Collectively, these pathways form a regulatory network that integrates both stimulatory and inhibitory inputs to fine‐tune Sox9‐driven differentiation.^39^

## 4 CHONDROGENESIS INDUCERS

Cartilage regeneration remains a significant clinical challenge due to the naturally avascular structure and low intrinsic healing capacity of cartilage tissue. A foundational mechanism for overcoming these limitations is chondrogenesis—the process through which stem or progenitor cells differentiate into chondrocytes that produce and organize cartilage matrix. This section reviews the principal inducers used to stimulate this process, including growth factors, small molecules, gene therapy, mechanical stimulation, and combination strategies in tissue engineering. Coordinating these interventions facilitates the regeneration of cartilage tissue and supports the advancement of regenerative therapies.

### 4.1 Growth factors

Chondrogenesis, the complex process of cartilage formation from progenitor cells, is intricately controlled by a suite of chondrogenesis inducers. These inducers include TGF‐βs, BMPs, FGFs, IGFs, and growth differentiation factor 5 (GDF‐5). Each of these molecules plays an essential role in directing the critical phases of chondrogenesis, namely, cell proliferation, differentiation, and ECM production. Table 1 lists chondrogenic growth factors by year of discovery, detailing their roles and the specific mechanisms through which they regulate the process.

**TABLE 1:** Chondrogenic growth factors listed by discovery year.

Name	Tested cell lines	Combination tested form	Mechanism	Year	References
Transforming growth factor‐β1	Embryonic chick limb cells, chick periosteum‐derived mesenchymal cells, C3H10T1/2, ATDC5, adipose‐derived adult stromal (ADAS) cells	Porous gelatin‐chondroitin‐ hyaluronate scaffold, chitosan scaffolds, PCL films, TGF‐β1‐loaded fibrin glue, macroporous poly(DL‐lactic‐co‐glycolic acid) (PLGA) sponges, loaded alginate bead	Via induction of pro‐survival Akt cascade and TIMP‐3	1989	41
Transforming growth factor‐β2	Human bone marrow‐derived mesenchymal progenitor cells	N/A	Via activating activin receptor‐like kinase 5/Smad3 signaling	1990	42
Growth differentiation factor 5	Rat limb bud cells, human mesenchymal stem cells, ATDC5 cells, chick embryonic limb mesenchymal cells	GDF5‐conjugated BMSC‐laden hydrogel and polymer, hierarchical porous ECM scaffold incorporating GDF‐5	Through p38 MAP kinase signaling	1996	43
Bone morphogenetic protein‐7	Chick embryo sternal chondrocytes, rat calvaria‐derived chondrogenic C5.18 cells, human bone marrow multipotent mesenchymal stromal cells	BMP‐7‐loaded PLGA nanoparticles electrosprayed onto the scaffolds	Via Smad1/5 phosphorylation and Smad‐independent signaling	1997	44
Bone morphogenetic protein 4	Limb bud mesoderm cells, muscle‐derived stem cells, ATDC5	N/A	Via positively regulated by Sox9 and negatively by Msx2	1998	45
Bone‐morphogenetic protein‐2	Mouse primary chondrocytes and chondrocytic MC615 cells, C3H10T1/2, bovine synovium‐derived progenitor cells, human Achilles tendon‐derived stem cells	Perlecan domain I‐conjugated, hyaluronic acid‐based hydrogel particles, mineral‐coated hydroxyapatite microparticles (MCM)	Involves p38 mitogen‐activated protein kinase (MAPK)‐mediated down‐regulation of Wnt‐7a pathway	1998	46
Bone morphogenetic protein‐6	Termed human adipose‐derived adult stem cells (ADAS cells), human marrow stromal cells, ATDC5 cells	Chitosan scaffolds loaded with bone morphogenetic protein‐6	Through the Smads pathway and MAPK pathways	2001	47
Fibroblast growth factor‐18	Human bone marrow mesenchymal stem cells, adult articular chondrocytes	N/A	Via fibroblast growth factor receptor 3	2002	48
Bone morphogenetic protein‐9	C3H10T1/2, ATDC5 cells	N/A	Via Smad1/5 phosphorylation	2003	49
Connective tissue growth factor	Rat condylar chondrocytes, auricular chondrocytes	N/A	Via PI3K/Akt pathway	2004	50
Insulin‐like growth factor‐1	ATDC5 cells, chick limb bud mesenchymal cells	Coacervate‐embedded composite hydrogels, hyaluronic acid binding	Via ERK activation	2004	51
Transforming growth factor‐β3	Human bone marrow‐derived mesenchymal stem cells, rabbit chondrocytes	Microspheres, thermo‐reversible hydrogel, injectable hydrogels	Via the Smad‐dependent pathway and a non‐Smad pathway)	2006	52

Abbreviations: BMP, bone morphogenetic proteins; BMSC, bone marrow stromal cells; ERK, extracellular signal regulated kinases; PCL, polycaprolactone; PI3K; phosphoinositide 3‐kinase; TGF‐β, transforming growth factor beta.

TGF‐β isoforms, particularly TGF‐β1 and TGF‐β3, are central to chondrogenesis. TGF‐β1 facilitates the condensation of pre‐cartilage mesenchymal cells, a crucial precursor event leading to chondrocyte differentiation. Conversely, TGF‐β3 stimulates mesenchymal cell proliferation, further aiding early cartilage formation. These isoforms are typically applied at concentrations of around 10 ng/mL, with exposure durations of 3–4 weeks to optimize their chondrogenic effects.^53^ Similarly, BMPs, particularly BMP‐2 and BMP‐7, are potent inducers of chondrogenic differentiation. Shintani et al. demonstrated that BMP‐2 promotes ACAN expression in MSCs in a dose‐dependent manner, with concentrations ranging from 200 to 2000 ng/mL over a 4‐week period.^54^ In parallel, Knippenberg et al. reported that BMP‐7 induces ACAN expression at a dose as low as 10 ng/mL within 14 days.^55^ However, both BMP‐2 and BMP‐7 were also found to upregulate Runx2, a transcription factor that positively regulates osteogenesis, alongside Sox9 expression.^55^, ^56^ This dual induction poses a significant challenge for targeting BMPs in cartilage regeneration: while BMPs promote chondrogenesis, they may concurrently activate osteogenic pathways, reducing their specificity in stem cell‐based repair strategies.

Fibroblast growth factor‐18 (FGF‐18) also plays a crucial role in chondrogenesis by enhancing chondrocyte proliferation and differentiation, thus promoting cartilage formation.^57^, ^58^ Its ability to stimulate anabolic activities in chondrocytes has shown significant promise in therapeutic applications for cartilage repair and regeneration. Additionally, insulin‐like growth factor 1 (IGF‐1) is vital for chondrogenesis, promoting chondrocyte proliferation, matrix production, and inhibiting chondrocyte apoptosis.^51^, ^59^ It also promotes the proliferation and chondrogenic differentiation of MSCs, underscoring its importance in cartilage tissue engineering. GDF‐5, a member of the BMP family, is similarly significant in chondrogenesis, enhancing chondrocyte proliferation and differentiation while supporting matrix production.^43^ Its role in the p38 mitogen‐activated protein kinase (MAP) pathway highlights the involvement of GDF‐5 in the complex signaling networks that regulate chondrogenesis.^43^, ^60^

A number of published studies have revealed the intricate mechanisms through which these growth factors promote chondrogenesis. For example, TGF‐β1 induces the pro‐survival Akt cascade and upregulates TIMP‐3, while BMP‐7 promotes chondrogenesis via Smad1/5 phosphorylation and Smad‐independent pathways.^61^, ^62^, ^63^ The involvement of the p38 MAP kinase pathway, especially through GDF‐5, further emphasizes the complexity of chondrogenic signaling networks.

Despite the promise of these chondrogenesis inducers, several limitations must be addressed. The effectiveness of factors like BMPs and TGF‐β is highly dose‐dependent, with the risk of adverse effects such as unintended osteogenic differentiation leading to ectopic bone formation.^46^, ^55^, ^64^ Proper temporal and spatial regulation of growth factor delivery is crucial, while incorrect timing or localization can lead to premature differentiation or inadequate matrix production. Additionally, growth factors typically have a short half‐life in the body, meaning they degrade quickly and lose their effectiveness.^65^ This short half‐life, combined with the rapid diffusion of growth factors away from the target site, makes it challenging to maintain a high concentration where it is needed. High levels of proteolytic enzymes in the body can further degrade these factors, reducing their stability. The transient nature of growth factor effects necessitates the development of sustained delivery systems, which are challenging to create and may not ensure consistent long‐term release. High production costs and technical difficulties in achieving the necessary purity and scale further limit widespread application. Finally, ethical and regulatory considerations, particularly concerning growth factors derived from human or animal sources, pose additional challenges that may slow the development and approval of these therapies.

### 4.2 Small molecule inducers

Small molecules can serve as a straightforward and efficient means to enhance cell proliferation, maintain a consistent chondrocyte phenotype, and facilitate stem cell differentiation into chondrogenic lineages.^66^ Moreover, the preservation and practicality of these small molecules are easier compared to protein‐based factors, making them more suitable for long‐term storage, transportation, and clinical applications.^67^ Their stability and ease of handling contribute to their growing appeal in both research and therapeutic contexts focused on cartilage regeneration and repair. Supplementary Table S1 summarizes the small molecules that induce chondrogenesis, highlighting the year of discovery, mechanisms of action, and the cell lines used for testing.

Three prominent chondrogenesis activators, including KGN, TD‐198946, and melatonin, have been reported to improve chondrogenic differentiation of stem cells. Firstly, KGN induces chondrogenesis by displacing core‐binding factor beta (CBFβ) from filamin A and relocates to bind with RUNX1; therefore, it enhances the synthesis of cartilage matrices, such as Col2A1 and ACAN.^68^ KGN has been employed in various forms, including intra‐articular injections, integration with growth factors, incorporation into drug delivery systems, and combination with scaffolds.^69^, ^70^, ^71^ The second candidate is TD‐198946, a thienoindazole derivative found to be a potent chondrogenic agent in 2012. It has been shown to enhance glycosaminoglycan synthesis in nucleus pulposus cells and prevent intervertebral degeneration.^72^ TD‐198946 effectively promotes chondrogenic differentiation while avoiding hypertrophy in both cells and metatarsal organ cultures.^73^ In addition, TD‐198946 has been reported to enhance chondrogenic induction from human synovium‐derived stem cells through the NOTCH3 signaling pathway.^74^ The mechanism of action of TD‐198946 mainly occurs via the PI3K/Akt signaling pathway.^72^ Furthermore, TD‐198946 has been processed and applied in intra‐articular injection.^73^, ^75^ Lastly, melatonin, an endocrine hormone primarily secreted by the pineal gland, was shown to play a significant role in the process of cartilage formation from MSCs. It enhances chondrogenic differentiation of human MSCs by upregulating the expression of critical genes involved in this process, including ACAN, Col2A1, Col10A1, Sox9, and RUNX2.^76^ Melatonin also increases the synthesis of glycosaminoglycans (GAG), a crucial component of the ECM in cartilage. These effects are at least partially mediated through melatonin receptors, as demonstrated by the partial blocking of these effects by luzindole, a melatonin receptor antagonist.^76^ It was also reported that the melatonin effect on chondrogenic differentiation of MSCs is influenced by the Wnt signaling pathway.^77^ This means the concentration of melatonin can influence its effects. High concentrations may inhibit the proliferation and differentiation of chondrocytes, while moderate concentrations may be beneficial.^78^

### 4.3 Gene therapy

The concept of reprogramming cells within the joint, including MSCs, through gene transduction to confer beneficial functions such as modulating chondrogenesis, thus promoting MSCs differentiation into chondrocytes by producing collagen type II and proteoglycans, holds potential for addressing cartilage regeneration and other pathologies. In gene therapy for chondrogenesis, specific genes encoding key regulators of cartilage formation, such as growth factors (IGF‐1, TGF‐β, and BMPs) or transcription factors (Sox9), are delivered to target cells to enhance their chondrogenic potential. Transferring a gene to the cell or organ requires a vector or multiple vectors, which can be either non‐viral or viral. The process of transferring genes using non‐viral vectors is referred to as transfection, whereas gene transfer via viral vectors is known as transduction.

In terms of non‐viral vectors, the expression vector (plasmid) is commonly used as liposomes or others like chemical, electrical, and mechanical methods. On the other hand, commonly used viral‐based vectors include adenoviruses, recombinant adeno‐associated viral (rAAV), retroviruses, and baculoviruses. Bone marrow was transduced with rAAV‐FLAG‐hsox9, resulting in enhanced duration of matrix biosynthesis and chondrogenic activities compared to the control group.^79^ In 2018, lentivirus was effectively utilized as a vector to deliver TGF‐β1 into bone marrow stem cells for transduction, which improved the repair of the cartilage defect.^80^

Compared to recombinant protein replacement treatment with short half‐lives, gene‐based therapies offer longer‐lasting, targeted expression of proteins in a more physiologically relevant manner. However, this approach faces challenges, including the difficulty of integrating therapeutic DNA into target cells and the risks of immune responses and toxicity.^81^

### 4.4 Mechanical stimulation

The fate of stem cells is influenced by more than just soluble factors and cell–cell interactions. It is also regulated by the physical environment, which is characterized by distinct mechanical and chemical properties. These environmental factors play a crucial role in regulating numerous intracellular processes and directing cell behavior. Mechanical stimulation is pivotal in chondrogenesis and cartilage development, as both genetic and cellular signals work together to physically shape and pattern tissues.

Chondrocytes are enveloped by a thin layer called the pericellular matrix (PCM) that absorbs forces and transfers them to the cell surface. Recent research indicates that molecules like perlecan, collagen, and hyaluronan not only give the PCM its unique physical properties but also help maintain the microenvironment around chondrocytes.^82^, ^83^ These molecules transmit physical signals detected by receptors on the cell membrane, including calcium channels, primary cilia, integrins, and cell–matrix interactions, activating downstream signaling cascades like MAPK, Rho/ROCK, and PI3K/Akt. This initiates a series of molecular signaling pathways that allow chondrocytes to respond to mechanical stresses by converting the physical signals into chemical and biological signals that regulate gene transcription.

Mechanical factors should be considered throughout the entire process of articular cartilage regeneration, from the initial cultivation of seed cell cultures to the subsequent reconstruction of 3D cartilage tissue. Biomechanical cues associated with cartilage development, such as tension, hydrostatic pressure, shear, and compression, have been shown to impact chondrogenic differentiation.^84^ Studies have illustrated that mechanical stimulus, such as dynamic compression and hydrostatic pressure, can increase the expression of chondrogenic genes and promote the synthesis of ECM components by MSCs.^84^, ^85^, ^86^, ^87^ Furthermore, mechanical stimulation has been found to modulate the secretory profile of MSCs, potentially enhancing their paracrine activity and promoting tissue regeneration.^88^

The viscoelastic properties of the PCM give rise to the highly heterogeneous mechanical environment surrounding chondrocytes.^89^ In situ imaging studies and zone‐specific finite‐element models of cell–matrix interactions in cartilage revealed the biomechanical role of the PCM in regulating local stress, strain, and fluid flow environments.^90^ Dynamic compressive loading enhances chondrogenic gene expression, matrix synthesis, and mechanical properties of MSC‐seeded constructs.^12^ Optimal conditions include low frequency (~1 Hz), moderate strain (10%–15%), and longer duration.^91^, ^92^, ^93^ Hydrostatic pressure (0.1–10 MPa) stimulates chondrogenesis by regulating osmotic pressure, fluid flow, streaming potential, proteoglycan synthesis, and enhances proteoglycan synthesis.^94^ Fluid shear stress promotes chondrogenic differentiation by modulating calcium signaling and gene expression.^95^ While low‐shear stresses promote chondrogenesis, high‐shear stresses can be catabolic. Tensile loading stimulates chondrogenesis but can also induce hypertrophy at high strains, especially in superficial zone chondrocytes, which are more sensitive. To mimic the in vivo joint environment better, multimodal bioreactors that combine compression, shear, and hydrostatic pressure are being developed.^96^

### 4.5 Combinatory approach in tissue engineering

**FIGURE 4:**
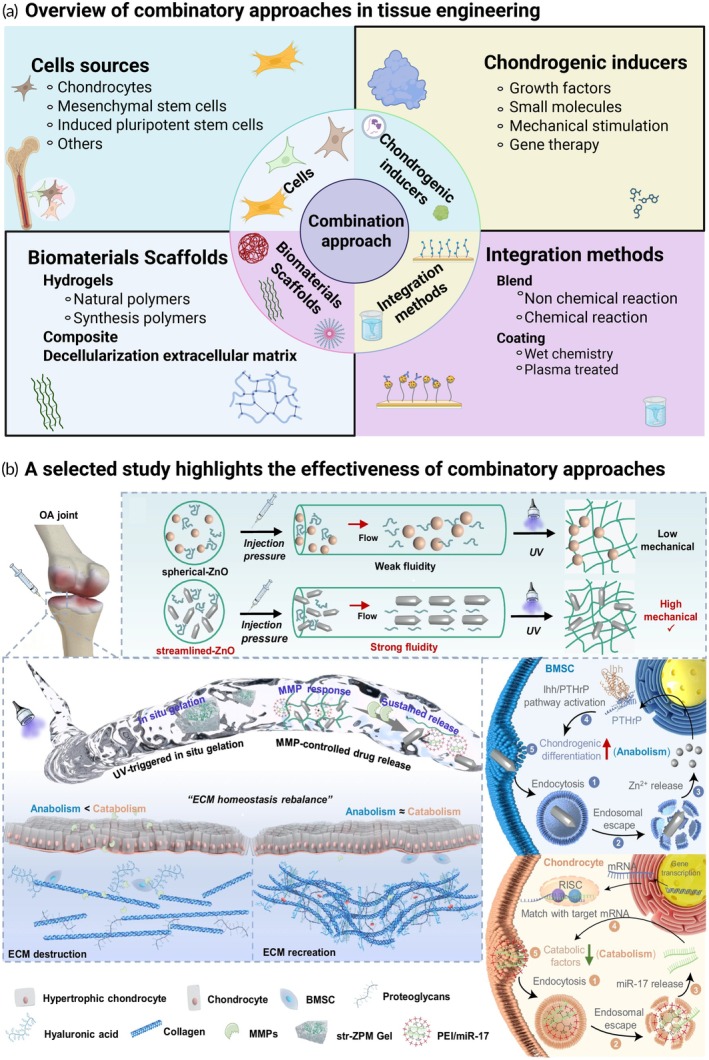
Combinatory approaches in tissue engineering for cartilage regeneration. (a) Provides an overview of key elements applied in cartilage‐directed tissue engineering. Cell sources include chondrocytes, mesenchymal stem cells, and induced pluripotent stem cells. Chondrogenic inducers comprise growth factors, small molecules, mechanical stimuli, gene therapies, and extracellular vesicles. Biomaterials span natural and synthetic hydrogels as well as composites derived from decellularized extracellular matrix. Integration techniques involve component blending through chemical or non‐chemical interactions, and surface coating via wet chemistry or plasma‐based methods. (b) A combinatory approach integrating miR‐17 and streamlined ZnO within an engineered GelMA‐based hydrogel. The construct mimics native extracellular matrix (ECM) properties, supporting cell proliferation, migration, and adhesion, while demonstrating favorable biocompatibility and biodegradability. The streamlined ZnO structure enhances hydrogel fluidity and mechanical integrity, enabling in situ injectability and sustained release through gradual degradation. Internalization by bone marrow stromal cells (BMSCs) and chondrocytes leads to localized Zn^2+^ release, which activates the Indian hedgehog/parathyroid hormone‐related protein (Ihh/PTHrP) signaling axis, promoting BMSC recruitment and chondrogenic differentiation. Concurrently, miR‐17 attenuates catabolic damage by suppressing MMP13 and ADAMTS5 expression. Together, these coordinated actions rebalance anabolic and catabolic dynamics within the ECM, ultimately supporting cartilage protection and restoration of joint function. This figure was constructed using BioRender and adapted from “Streamlined metal‐based hydrogel facilitates stem cell differentiation, extracellular matrix homeostasis and cartilage repair in male rats” by Li et al.^97^ OA, osteoarthritis; PEI, polyethylenimine; RISC, RNA‐induced silencing complex; UV, ultraviolet radiation.

Combinatory approaches in tissue engineering that integrate biomaterials and chondrogenesis inducers are potent pathways to enhancing cartilage regeneration. Cartilage has a highly specialized structure that demands both mechanical strength and biocompatibility to function effectively. Its mechanical strength is crucial for joint movement and weight‐bearing, as it must endure compressive forces, shear stress, and continuous mechanical loading throughout life. To replicate these characteristics, various biomaterials have been explored for cartilage tissue engineering for the delivery of chondrogenesis inducers, each with distinct advantages in mimicking the mechanical and biological properties of natural cartilage. A comprehensive strategy for chondrogenic tissue engineering typically rests on two interdependent components: inductive cues and engineered scaffolds (Figure 4a). Bioactive cues, including growth factors, small molecules, mechanical stimuli, and gene vectors, play a central role in directing mesenchymal stem cell differentiation and stimulating ECM synthesis. In parallel, biomaterial scaffolds—including hydrogels (derived from natural or synthetic polymers), composites, and decellularized cartilage tissue—provide a 3D framework that supports cell attachment, guides tissue architecture, and fosters long‐term phenotypic stability during maturation. Hydrogels, with their high‐water content, provide a 3D environment similar to the native cartilage ECM for promoting cell encapsulation and nutrient diffusion.^98^, ^99^ The high level of hydration is crucial for replicating cartilage resistance to compression, as water is a major component of the ECM.^100^, ^101^ Polymers, both natural (e.g., collagen and chitosan) and synthetic (e.g., polyvinyl alcohol and polyethylene glycol), are engineered to mimic cartilage mechanical properties, offering flexibility, strength, and biodegradability. Natural polymers, such as collagen, replicate the ECM composition, while synthetic polymers like polyvinyl alcohol (PVA) allow for tailored mechanical strength and controlled degradation rates.^102^ Composites combine different materials, such as polymers and ceramics, to optimize mechanical strength and biological properties.^103^ For example, incorporating hydroxyapatite or calcium phosphate into composites enhances mechanical strength while maintaining biocompatibility.^104^ Decellularized cartilage, as a natural scaffold, retains the tissue‐specific architecture and biochemical signals of native cartilage, offering an ideal environment that supports cellular infiltration and differentiation while promoting cartilage repair.^105^

To enhance the chondrogenic potential of these biomaterials, various methods are employed to incorporate chondrogenic inducers. One common approach is blending, where chondrogenic factors are blended into the biomaterial during fabrication, ensuring controlled and sustained release. This method is particularly suitable for hydrogels, allowing inducers to be mixed before crosslinking, facilitating slow and controlled release at a designed rate to promote effective chondrogenesis.^106^ For example, an ultra‐durable cell‐free bioactive hydrogel was developed with dual release of KGN and tannic acid for cartilage regeneration.^107^ Another method involves the use of surface coating, where chondrogenic molecules are applied to the surface. Traditional wet chemistry for the immobilization of chondrogenic molecules is often used, but these show some drawbacks such as substrate dependence, non‐uniform coatings, weak binding, and side‐reactions.^108^, ^109^ Coatings prepared via plasma technology can solve some of these drawbacks. The process is largely substrate independent, meaning the same coating can be applied on a variety of substrate materials, ensuring reproducible surface properties, distribution of immobilized molecules, and biocompatibility.^110^, ^111^, ^112^, ^113^, ^114^, ^115^ In 2023, the first study describing covalent protein binding on microfiber meshes for cartilage regeneration highlighted the significant potential of plasma immobilization in binding chondrogenic inducers to promote cartilage differentiation.^116^ Layer‐by‐layer (LbL) assembly is another approach that allows for the application of alternating layers of charged polymers, offering precise control over the type and quantity of factors incorporated.^117^ This technique enables the creation of growth factor gradients, simulating the natural heterogeneity of cartilage ECM.^117^ These strategies facilitate the creation of a controlled microenvironment that promotes chondrogenic differentiation, guiding stem cells toward cartilage formation and enhancing tissue regeneration.

Since 2023, cartilage tissue engineering has increasingly focused on combination strategies to enhance chondrogenesis. A notable example is the injectable piezoelectric hydrogel developed by Vinikoor et al., which converts ultrasound‐driven mechanical stimulation into localized electrical signals, promoting stem cell migration, chondrogenic marker expression, and effective cartilage regeneration.^118^ In 2024, Guo et al. developed a dynamic proteinaceous hydrogel capable of recruiting stem cells and TGF‐β1 to the site of cartilage defects, significantly promoting regeneration.^119^ Another noteworthy study published in late 2024 demonstrated promising cartilage regeneration using a blended chitosan hydrogel incorporating three growth factors—TGF‐β3, IGF‐1 (encapsulated as microspheres), and PDGF‐BB.^120^ This multifactorial system demonstrated strong in vitro and in vivo bioactivity, supporting coordinated and sustained cartilage repair.

In early 2025, a nanoparticle‐based platform was proposed to simultaneously deliver growth factors and gene therapy agents. Copper–mesoporous silica nanoparticles (CuO@MSN) co‐delivered BMP‐7 protein and a Sox9 plasmid, facilitating chondrogenic differentiation while suppressing hypertrophic transition in MSCs.^121^ Another study reported a silk‐based delivery system incorporating KGN, dexamethasone (DEX), and a BMSC‐specific affinity peptide. This environmentally friendly platform was demonstrated to enhance mechanical strength and allowed for sequential, sustained drug release.^122^

An illustrative example of this integrative approach comes from a recent study by Wen et al., who developed a biomimetic hydrogel system (GelMA/str‐ZnO@PEI (polyethylenimine)/miR‐17) designed to replicate native cartilage properties while supporting mechanical loading (Figure 4b).^97^ The incorporation of ZnO nanostructures enhanced the hydrogel's injectability and mechanical resilience. Following intra‐articular administration, the hydrogel underwent gradual degradation, releasing Zn^2+^ ions that activated the Ihh/PTHrP signaling axis, facilitating BMSC recruitment and chondrogenic differentiation. Concurrently, the sustained release of miR‐17 downregulated catabolic enzymes such as MMP13 and ADAMTS5, thereby preserving ECM integrity and contributing to functional joint restoration. This dual‐action platform highlights the therapeutic potential of combining biochemical and genetic cues within a bio‐responsive scaffold for targeted cartilage repair in osteoarthritic conditions.

## 5 METHODS FOR EVALUATION OF CHONDROGENESIS

Various in vitro, in vivo, and clinical assessments have been used to achieve a multifaceted understanding of the processes involved in chondrogenesis. In vitro studies have been instrumental in investigating the chondrogenic potential of various cell types and novel scaffolds. These offer opportunities to generate early insights into cell behavior and tissue formation under controlled microenvironments, cost‐effectiveness, high throughput, and reduced animal use. However, challenges arise in translating in vitro findings to clinical success, as studies have shown inconsistencies between in vitro chondrogenic capacity and clinical outcomes.^123^

Preclinical assessments are crucial in understanding the potential advantages and challenges of using chondroprogenitors in stem cell therapy. As a specialized group of multipotent progenitors, chondroprogenitors offer advantages such as being more committed to the chondrocyte lineage and potentially reducing chondrocyte dedifferentiation rates. Despite challenges in identifying practical and abundant sources of expandable chondroprogenitors, their use in stem cell therapy presents clear benefits such as clonability and commitment to the chondrogenic lineage.^124^

Integrating insights from in vivo, in vitro, and clinical assessments offers a comprehensive understanding of cartilage regeneration processes and enhances the translation of research outcomes into effective clinical interventions. These evaluation methods provide valuable insights into the advancement of chondrogenesis, highlighting both the challenges and opportunities in bridging the gap between research findings and clinical applications.

### 5.1 In vitro models

When selecting a cell line for chondrogenesis evaluation in vitro, numerous factors need to be considered, including differentiation potential and relevance to the human condition. In this context, human MSCs, including human bone marrow stem cells, human adipose‐derived stem cells, and human umbilical cord‐derived stem cells, are more popular choices due to their high chondrogenic potential and well‐established differentiation protocol.^125^ Despite this, the application of MSCs still encounters considerable challenges, such as cellular aging and donor‐dependent differences. Induced pluripotent stem cells (IPS) may offer a suitable alternative with high potential, especially for modeling cartilage diseases and creating patient‐specific tissues.^126^, ^127^ Although human cell culture is costly, mouse‐derived cell lines such as ATDC5 and C3H10T1/2 offer cost‐effective alternatives with established chondrogenic differentiation potential. These models have been widely used to investigate cartilage differentiation, matrix deposition, and lineage commitment.^128^, ^129^ However, species‐specific differences in gene regulation, signaling dynamics, and phenotypic outcomes can significantly affect the results' translatability. For instance, orthologous genes often display divergent expression profiles between mouse and human cells, particularly in pathways involved in skeletal development and immune modulation.^130^, ^131^ These interspecies differences may compromise the predictive utility of murine cell models in cartilage regeneration and skeletal developmental research. In contrast, human cells better reflect native biological processes and offer higher fidelity when translating findings to clinical settings. Yet, their use in vivo typically requires immunosuppressed mouse models or humanized systems to prevent xenogeneic rejection during transplantation.^132^ These practical and biological constraints necessitate a strategic balance between affordability, experimental compatibility, and translational accuracy when choosing cell models for cartilage regeneration research.

**FIGURE 5:**
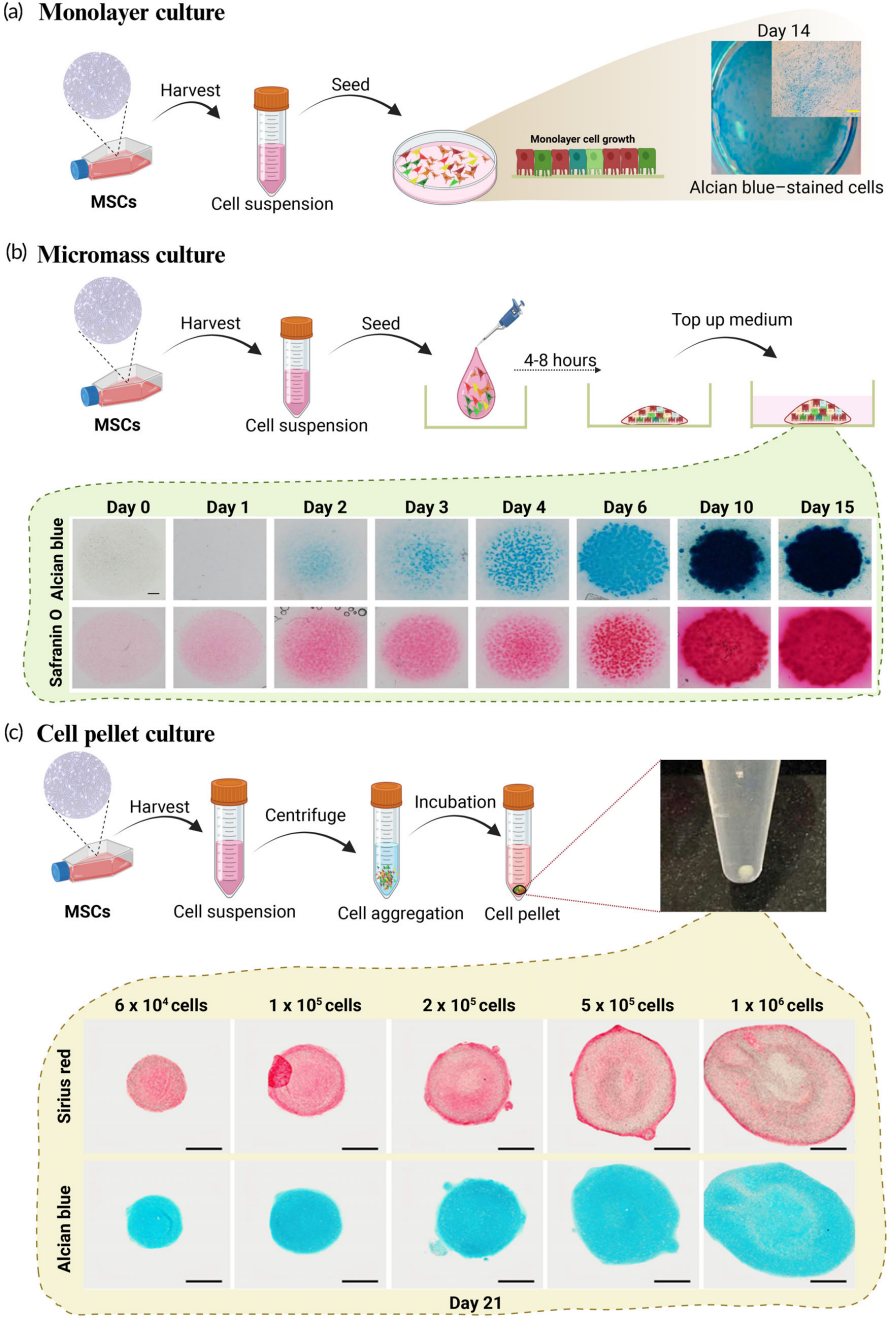
Staining cartilage differentiation in different in vitro models. (a) In the monolayer culture model, mesenchymal stem cells (MSCs) are directly seeded onto cell culture plates, distributing uniformly across the wells. This setup allows for cell expansion and supports subsequent in vitro cartilage differentiation assays. (b) In micromass culture, additional steps are required. Cells are deposited at the center of each well at high density; once adherent, culture medium is added. Cartilage matrix production is assessed through Alcian blue and Safranin O staining after a series of culture days. (c) The cell pellet culture involves further processing compared to monolayers. Cells are centrifuged to promote aggregation, forming a three‐dimensional construct. After 21 days of culture, matrix deposition is visualized using sirius red and Alcian blue staining, with results varying by initial cell number. Figure adapted from multiple sources under Creative Commons Attribution License.^135^, ^136^, ^137^

While monolayer cultures offer a straightforward method for cell expansion and manipulation, they frequently result in diminished production of key cartilage proteins such as glycoproteins and Col2A1, and cells will undergo dedifferentiation after 2 weeks.^133^, ^134^ As shown in Figure 5a, this method involves culturing MSCs in a two‐dimensional environment for 14 days, followed by Alcian blue staining to detect the proteoglycan‐rich matrix, which serves as a crucial indicator of chondrogenic differentiation. While convenient and widely used, this model fails to replicate the complex cell–matrix interactions of native cartilage tissue. In contrast, 3D cultures such as cell pellets, micromass, or hydrogels are commonly preferred in vitro models for chondrogenesis evaluation because they closely replicate the native tissue environment and promote enhanced expression of cartilage‐specific markers.

To address these limitations, high‐density cell culture systems (3D), including cell pellets, micromass cultures, or hydrogels are utilized. These systems create a 3D environment that preserves the chondrocyte phenotype and facilitates the development of structures resembling cartilage. As illustrated in Figure 5b, the micromass culture method involves seeding a high‐density droplet of MSCs, which aggregate to form a condensation‐like zone that promotes chondrogenic differentiation. Over 15 days, ECM production is assessed through Alcian blue and Safranin O staining, revealing progressive deposition of glycosaminoglycans and proteoglycans—key components of the cartilage ECM. In addition, cell pellets are a simple yet effective method for enhancing chondrogenic differentiation by promoting increased cell‐to‐cell interactions. Hydrogels serve as scaffolds mimicking the mechanical properties of cartilage and are able to provide mechanical stimuli to enhance chondrogenesis.^138^ These 3D models are crucial for generating physiologically relevant data and advancing our understanding of cartilage formation and regeneration.

The cell pellet culture method is widely employed to induce chondrogenesis in vitro, as it closely replicates the native microenvironment of chondrocytes by facilitating high cell contact and condensation, as demonstrated in Figure 5c. To initiate this process, samples containing one to 500,000 cells are suspended in a small volume of chondrogenic medium and then centrifuged. Subsequently, the resulting cell pellets are placed in loosely capped tubes and incubated in a cell incubator. After 24 h, the cells settle at the bottom of the tube, forming spherical aggregates. The culture medium was refreshed once every 3 days, and the cell pellets were collected after a period of incubation.^139^ Figure 5c demonstrates how initial cell seeding density influences pellet culture morphology and matrix organization over a 21‐day period. Pellet size increases with higher MSC numbers, from 6 × 10^4^ up to 1 × 10^6^ cells per pellet. However, excessive cell density appears to compromise matrix architecture, as aggregates seeded with 1 × 10^6^ cells show less compact distribution of ACAN and reduced matrix condensation compared to those with lower starting densities. These findings underscore the importance of optimizing initial cell numbers to balance aggregate growth with matrix quality in 3D chondrogenic models. This method helps cells aggregate (the first step into chondrogenesis), which also helps stabilize the chondrogenic potential of the culture cells. A prevalent issue arises where cells within the central region of the pellet are frequently discovered to be undifferentiated or necrotic, with only those cells in direct contact with the conditioning medium undergoing chondrogenic differentiation.^140^ In addition, MSCs tend to differentiate into fibrocartilage‐like tissue with an increase of collagen I and hypertrophy cartilage (collagen X). The size of pellets can vary from 1 to 2 mm in diameter depending on the specific application, and the weight from 200 to 300 mg.^140^

In 2007, micromass was applied to promote the chondrogenic differentiation of human mesenchymal stem cells (hMSCs) by Scharstuhl et al.^139^ Initially, the cells were collected and resuspended in the chondrogenic medium at a specific concentration. Subsequently, small droplets of the cell suspension were placed in a multi‐well cell culture plate, and after allowing the cells to adhere, an additional chondrogenic medium was added. After a day, the cell droplets aggregated into micromasses, which were subsequently collected on the 7th, 14th, and 21st days for further analysis.^139^ Then, in 2010, Liangming and his colleagues compared micromass and pellet culture systems. The results showed that micromass culture had better‐induced cartilage differentiation tissue with a weight from 2 to 4 mg. Moreover, the micromass culture system had more homogenous and higher collagen II but lower collagen I and hypertrophic chondrocyte markers (collagen X). However, there was evidence of some loss of matrix in micromass pellets after 21 days.^141^

In summary, the micromass system provides a rapid screening tool to examine chondrogenic competence. In contrast, the cell pellet system creates a more physiologically relevant 3D environment to study complete chondrogenesis over time. The choice depends on the stage of differentiation to be analyzed and the research question. Combining these two approaches can provide comprehensive insights into chondrogenic processes.

### 5.2 In vivo animal models

Despite promising findings regarding chondrogenesis inducers in in vitro models, rigorous testing in animal models is essential before clinical introduction. In vivo models offer significant advantages over in vitro systems for evaluating cartilage differentiation and regeneration. One key advantage is that in vivo models allow cells and tissues to mature within a physiologically relevant human environment, experiencing natural biomechanical forces and biochemical cues from surrounding tissues. However, selecting the appropriate animal model(s) for assessing a specific product can be challenging due to the lack of an ideal animal model that perfectly replicates articular cartilage.^142^ This challenge is further compounded by ethical considerations surrounding the use of animals in research.

**FIGURE 6:**
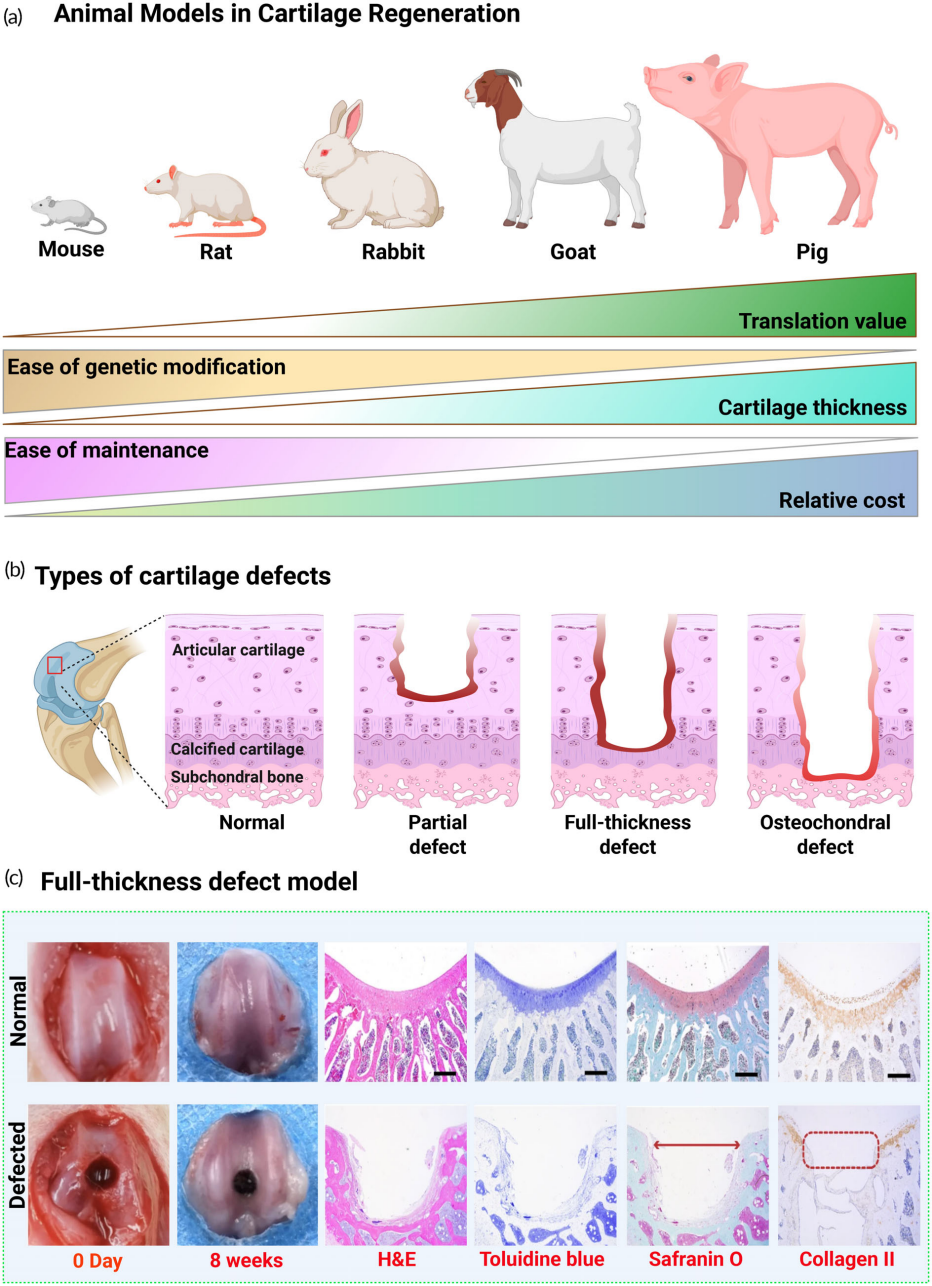
Overview of animal models and cartilage defect types in regeneration research. (a) Animal models commonly employed in cartilage regeneration are compared across four criteria: translational relevance, ease of genetic modification, cartilage thickness, and relative cost. Illustrated species include mouse, rat, rabbit, goat, and pig, with graphical bars indicating each model's strengths and limitations. (b) Cartilage defect types are schematically represented: Normal: intact cartilage and subchondral bone, partial defect: superficial damage limited to the cartilage layer, Full‐thickness defect: damage extends through the cartilage to the tidemark, osteochondral defect: involves both cartilage and underlying subchondral bone. (c) Full‐thickness defect model analysis displays histological results from normal and defective cartilage at Day 0 and Week 8. Sections are stained using hematoxylin and eosin (H&E), toluidine blue, Safranin O, and collagen II, capturing matrix integrity and chondrogenic restoration over time. Figure was adapted from “Ultra‐durable cell‐free bioactive hydrogel with fast shape memory and on‐demand drug release for cartilage regeneration”.^107^

Rodent models, particularly mice and rats, are frequently utilized in preclinical studies of cartilage regeneration due to their affordability, well‐characterized genetics, and ease of handling. In contrast, large animal models such as dogs, goats, sheep, and pigs are employed less routinely but offer greater anatomical and biomechanical relevance to human articular cartilage. Each species presents specific advantages and constraints in terms of joint size, cartilage thickness, biological response, housing requirements, and translational potential, as illustrated in Figure 6a.

Small animal models, mainly rodents, continue to be invaluable in providing information about chondrogenesis and cartilage tissue regeneration.^143^ The most significant advantage of the small animal models is that they allow for using transgenic and knockout strains, which help assess multiple factors that control cartilage formation.^144^ For instance, transcriptional coactivator with PDZ (a protein‐protein interaction domain)‐binding motif (TAZ) floxed/floxed mice were used to investigate its role in chondrogenesis.^145^ In addition, mouse‐deficient T‐cell function is also used in implantation to remove the impact of the immune system on different species or new materials. Nonetheless, it lacks appropriate surrounding articular tissues.^146^ Moreover, small animal models allow for long‐term research compared to in vitro models. For example, rabbits have been extensively employed to evaluate cartilage regeneration, with experiments typically lasting up to 4 months, although a few studies have extended to a year.^143^ They can be effectively bred and managed in indoor facilities, which helps reduce the cost compared to larger animal models. One limitation is that the joints of mice and rats are smaller, with very thin cartilage composed of only a few cell layers. Mouse cartilage thickness is only 30 μm, while rats, rabbits, and goats are 166.5, 356.2, and 907.5 μm, respectively.^147^, ^148^ The small size of the joint and the thinness of the mice cartilage make it impractical, unfeasible, and insignificant to investigate the impacts of surgical implants in this model. The subcutaneous implant model has been developed to overcome the limitations of the mouse joint size and cartilage thickness, allowing for the evaluation of tissue‐engineered constructs.^138^ Therefore, horses, goats, and sheep are the most commonly large animal models for cartilage repair.^142^ Furthermore, intra‐articular implantations of materials and cells are also conducted.^149^, ^150^

While numerous animals can be selected for experiments, there are three primary in vivo cartilage defect models commonly employed to assess strategies for cartilage regeneration and differentiation. To evaluate the efficacy of regenerative strategies, three primary types of defect models are commonly employed: partial‐thickness, full‐thickness, and osteochondral defects (Figure 6b).^151^ Partial‐thickness defects involve localized damage restricted to the articular cartilage, preserving both the underlying calcified cartilage and subchondral bone. Full‐thickness defects traverse the entire articular cartilage layer and reach the calcified cartilage zone. Osteochondral defects extend through the cartilage and penetrate into the subchondral bone, thereby recapitulating the pathophysiology of more severe clinical injuries.

In the cartilage defect model, most frequently carried out in animals like rabbits, rats, or mice, a controlled‐sized and shaped piece of cartilage is removed from a joint, typically the knee or ankle. This creates a defect that mimics cartilage injuries in humans. It can be employed to test different techniques, such as implanting stem cells or biomaterials, to study if these can promote cartilage regeneration and fill the defect. As exemplified in Figure 6c, a full‐thickness cartilage defect was induced in rat knees. After 8 weeks, the defect site remained unrepaired, as indicated by the persistent void. Hematoxylin and eosin (H&E) staining revealed no fibrous tissue infiltration, suggesting minimal intrinsic repair capacity. Toluidine blue stained proteoglycan‐rich cartilage in light blue, but this signal was absent at the defect site, reflecting depleted ECM content. Safranin O and Fast Green staining differentiated tissue types: neocartilage was undetectable in the defect group, whereas Fast Green highlighted surrounding acidophilic bone. Collagen type II immunostaining, typically visualized as brown layers within cartilage zones, further confirmed the absence of neocartilage on the defect side. Subcutaneous implantation, on the other hand, is when cells or biomaterials are directly implanted under the skin of animals, most often rats or mice. It can be used to study how cells or materials interact with the body tissues and potentially differentiate into cartilage‐like structures. While not directly testing joint repair, this model provides valuable insights into the basic biological processes involved. Last, induced osteoarthritis models can mimic joint damage and disease progression, allowing testing to promote repair. These models are most established through surgical, chemical, or mechanical methods.^152^

### 5.3 Molecular methods

**FIGURE 7:**
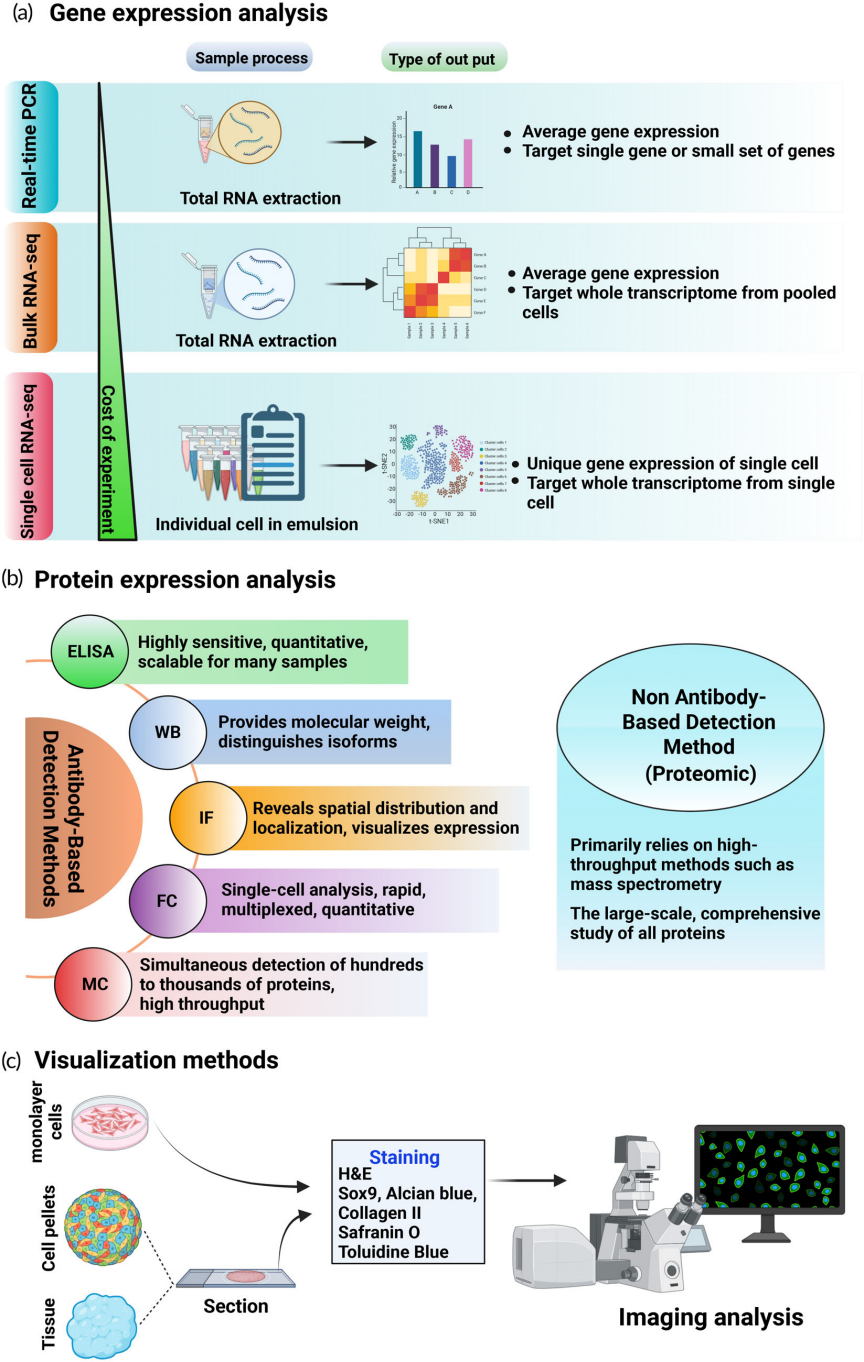
Common molecular techniques employed in the assessment of cartilage differentiation. (a) Gene expression analysis. Three platforms are illustrated with their sample workflows, output characteristics, and relative costs: real‐time polymerase chain reaction (PCR): uses total RNA to quantify the expression of select genes, low cost. Bulk RNA‐seq: extracts total RNA for transcriptome‐wide profiling across pooled cells. Moderate cost. Single‐cell RNA‐seq: captures individual cells in emulsion, enabling transcriptome profiling at single‐cell resolution. High cost. (b) Protein expression analysis. Includes both antibody‐based and proteomic strategies: antibody‐based: ELISA (quantitative, scalable), Western blot (isoform identification), immunofluorescence (spatial localization), flow cytometry (multiplexed and single‐cell), and mass cytometry (high‐throughput and high‐dimensional profiling). Non‐antibody proteomics: high‐throughput mass spectrometry for global protein analysis. (c) Visualization techniques. Workflows include the preparation of monolayer cells, cell pellets, or tissue, followed by sectioning. Common stains include hematoxylin and eosin (H&E), Sox9, Alcian blue, collagen II, Safranin O, and toluidine blue. Imaging is performed via microscopy combined with digital analysis. This figure was constructed using BioRender. ELISA, enzyme‐linked immunosorbent assay; FC, flow cytometry; IF, immunofluorescence; MC, protein microarrays; WB, western blot.

In the study of chondrogenesis in vitro and in vivo, various molecular methods are employed to examine any change during the chondrogenesis process. Gene expression is the first consideration method because the gene expression change is related to the chondrogenesis process, which appears early during chondrogenesis induction. For example, Sox9 and Col10A1 expression increases within the first 7 days of induction and then decreases, followed by Col2A1 expression later.^153^ During chondrogenic differentiation, the upregulation of key cartilage‐associated genes, including ACAN, Sox9, Col2A1, and Col10A1, serves as a molecular signature indicative of cartilage‐specific commitment. Figure 7a highlights three widely used techniques for gene expression analysis: real‐time polymerase chain reaction (PCR), bulk RNA sequencing, and single‐cell RNA sequencing. Each method varies in its target resolution, sample preparation, experimental cost, and the nature of its resulting output. Real‐time PCR offers exceptional sensitivity and specificity, allowing researchers to detect even small changes in mRNA levels. However, it requires dedicated primers for each gene target; its overall cost remains lower than that of broader transcriptomic techniques. In addition, bulk RNA sequencing is used for a comprehensive view of the transcriptome, although it averages gene expression across a population of cells.^154^ While technically challenging, single‐cell RNA sequencing can reveal heterogeneity within a cell population.^155^ As ribosomes translate mRNA transcripts into proteins in response to changes in gene expression, examining both protein and gene expression provides comprehensive evidence of chondrogenic differentiation. Protein analysis techniques such as western blotting and immunofluorescence are widely employed in chondrogenesis studies. Figure 7b highlights a range of protein detection methods, including antibody‐based assays such as the enzyme‐linked immunosorbent assay (ELISA), western blotting (WB), immunofluorescence staining, flow cytometry, and microarray platforms, as well as non‐antibody‐based techniques such as mass spectrometry‐based proteomics. Depending on the method, these assays can provide quantitative data, spatial localization, or high‐throughput protein profiling, facilitating a more comprehensive understanding of molecular events underpinning cartilage formation. While western blot is time‐consuming and requires a primary antibody against the protein of interest, it can semi‐quantify protein levels.^156^ On the other hand, immunofluorescence staining allows for the visualization of protein location within cells or tissues. Regarding the chondrogenesis process, it is necessary to detect the expression of Sox9, Col2A1, and ACAN, Col10A1, which are crucial proteins that contribute to cartilage differentiation. Histological examination provides essential insights into the morphology and structural organization of differentiated cells and tissues. Although the process is time‐consuming and demands specialized expertise, it remains a necessary component for validating cellular outcomes and tissue integrity. H&E staining provides an overview of tissue architecture and cellular morphology. For glycosaminoglycan detection, Safranin O, Alcian blue, and toluidine blue are commonly employed, while immunostaining for Col2A1 and Sox9 confirms chondrogenic differentiation at the molecular level, as illustrated in Figure 7c. While monolayer cultures may be stained directly, cell pellets and tissue samples require sectioning and mounting onto slides prior to histochemical evaluation. Histological staining techniques exploit distinct molecular affinities to visualize cartilage components. Cationic dyes such as Safranin O and toluidine blue bind selectively to negatively charged matrix constituents, particularly sulfated glycosaminoglycans, through electrostatic interactions.^157^, ^158^ In contrast, Alcian blue can stain a broader spectrum of acidic polysaccharides, including both sulfated and carboxylated glycosaminoglycans, depending on the pH of the staining solution. Dimethyl methylene blue (DMMB) is commonly used in spectrophotometric assays to quantify sulfated glycosaminoglycans in cartilage constructs, with absorbance shifts reflecting dye and GAG complex formation. For collagen visualization, Sirius Red staining, especially when combined with polarized light microscopy, enhances birefringence of fibrillar collagen (primarily type I), enabling spatial assessment of collagen fiber organization.^159^

To comprehensively evaluate chondrogenesis, recent studies have incorporated a range of advanced molecular and histological techniques. For instance, Yang et al. applied multiple modalities, including tissue staining with H&E, toluidine blue, Safranin O, and Collagen II. The team also used real‐time PCR to assess chondrogenic gene expression, which was subsequently confirmed at the protein level via western blotting.^107^ Similarly, Li et al. integrated immunohistochemistry, immunofluorescence staining, real‐time PCR, western blotting, and bulk RNA sequencing to probe cellular and molecular changes.^120^ A more recent comprehensive study further extended this approach by incorporating proteomic analysis, providing deeper insight into the evolving protein landscape associated with chondrogenic transformation.^160^ The consistent use of such multifaceted molecular strategies offers compelling evidence for the regenerative potential of the biomaterials under investigation.

These combined methodologies, encompassing gene expression analysis, protein quantification, and histological staining, have become increasingly standard in cartilage regeneration research. Their convergence provides a more robust, multidimensional understanding of regenerative outcomes and enhances the predictive power for clinical translation.

### 5.4 Clinical trials

Typically, the clinical development program for a new knee cartilage repair or replacement product should follow a structured sequence of phases, including early‐phase clinical studies and phase III or pivotal clinical studies. This orderly progression ensures the product is thoroughly evaluated before it is deemed safe and effective for general use.

Following the clinical study protocol from the U.S. Food and Drug Administration, clinical studies of articular cartilage repair or replacement products need to go through seven steps. These include the design, control group, study population, study efficacy endpoints, investigational product implantation procedures, follow‐up, and adverse experience reporting.^160^ It is important for early‐phase studies to gather data on safety, efficacy, and product characteristics.^161^ Control groups, such as placebo, sham surgery, active comparator, or standard care, are recommended for interpreting findings, particularly subjective outcomes like pain and function. Eligibility criteria for study participants should be clearly defined.

For phase III or pivotal studies, concurrent control groups, preferably randomized, are advised to ensure comparable populations. Primary endpoints should focus on clinically meaningful outcomes such as pain relief and improved physical function, with validated instruments. The International Knee Documentation Committee (IKDC) Subjective Knee Form, Knee Injury and Osteoarthritis Outcome Score (KOOS), Western Ontario and McMaster Universities Arthritis Index (WOMAC), and Lysholm scores are suggested for assessment.^162^ Secondary endpoints can include arthroscopic evaluation, physical examination findings, histological evaluation, serological assessments, and joint structure assessment by magnetic resonance imaging (MRI).

The study period should have a minimum follow‐up of 2 years for most products, with longer follow‐up for resorbed/degraded products and post‐market surveillance data collection. Detailed descriptions of surgical procedures for both investigational and control groups should be provided, and post‐operative care procedures should be standardized as much as possible. Adverse experiences should be reported systematically and categorized by type and relatedness to the product.

To gain approval for clinical use, products have to undergo extensive evaluations. Despite this, a product can also be withdrawn from the market after approval. For example, Invossa was previously approved in South Korea. It is a specialized therapy for osteoarthritis involving a combination of allogeneic non‐transformed chondrocytes (HC cells) and retrovirally transduced chondrocytes (TC cells) that overexpress TGF‐β1.^163^ However, the decision was later revoked because it contained an ingredient derived from a kidney instead of cartilage.^164^ Although there are numerous clinical trials annually, there are few approved products, including Cartistem, Bioseed‐C, CaReS, JACC, MACI, Novocart 3D, OrthoACI, Chondro‐Gide CE Mark, and HyaloFast CE Mark.^165^ Most of them combine autologous articular chondrocytes with biomaterials. Table S2 provides an overview of selected cartilage products engineered for chondrogenesis induction, including those that have received regulatory approval and those currently undergoing clinical trials.

An alternative approach that has not received formal U.S. Food and Drug Administration (FDA) approval is platelet‐rich plasma (PRP). However, PRP has been widely used for intra‐articular injections due to its regenerative potential and ability to promote cartilage repair.^166^, ^167^ Although PRP is not classified as a cartilage replacement product, its application in early‐phase studies has demonstrated promise in improving pain relief and supporting cartilage function, complementing other more traditional interventions.^168^, ^169^

## 6 PERSPECTIVE

The challenge of repairing cartilage damage is considerable, especially as natural repair mechanisms weaken with age. While current approaches like microfracture and implants offer temporary relief, achieving long‐term restoration remains challenging. There is, however, new hope in leveraging bioactive stimuli to enhance the innate chondrogenic potential and direct stem cells toward chondrocytes. Despite these promising developments, the translation of these innovations into clinical settings is hindered by significant barriers and inconsistent outcomes.

Recent breakthroughs in biomaterials are set to redefine the landscape of cartilage regeneration. Hybrid bio‐scaffolds that amalgamate chondrogenesis inducers with supportive microenvironments and stem cells are showing substantial promise in enhancing therapeutic efficacy and durability, as well as in facilitating their clinical adoption. These bioengineered biomaterials can act as versatile platforms that are capable of delivering genes, chemical agents, and growth factors that promote cartilage differentiation and repair, embodying a comprehensive strategy to improve tissue regeneration.

While these bioengineered biomaterials offer exciting prospects for advancing cartilage regeneration, it is essential to integrate innovation with rigorous evidence‐based practices. Tailoring strategic combinations of chondrogenesis inducers and materials to meet specific translational challenges—such as stability and minimizing off‐target effects—is vital. With a commitment to evidence‐based methodologies and tempered expectations, the potential for significant enhancements in patient outcomes through innovative biomaterials is achievable. The fusion of these advanced biomaterials with leading‐edge technologies marks the onset of a transformative era in cartilage regeneration, poised to substantially benefit those afflicted with cartilage injuries.

## AUTHOR CONTRIBUTIONS

Writing—original draft, conceptualization, and visualization: **Manh Tuong Nguyen**. Writing—review and editing: all authors. Supervision and validation: **Stan Gronthos**, **Yunpeng Zhao**, **Vashe Chandrakanthan**, **Vi Khanh Truong**, and **Krasimir Vasilev**. Resources: **Manh Tuong Nguyen** and **Krasimir Vasilev**. Investigation and project administration: **Krasimir Vasilev**.

## CONFLICT OF INTEREST STATEMENT

There are no conflicts of interest to declare. During the preparation of this work, the authors used ChatGPT and Perplexity to improve readability and language. After using these tools, the authors reviewed and edited the content as needed and took full responsibility for the content of the publication.

## Supporting information


**Table S1.** Chondrogenesis‐inducing compounds by year of discovery.
**Table S2.** Selected cartilage regeneration products.

## Data Availability

Data sharing not applicable to this article as no datasets were generated or analysed during the current study.
