# Supplementary material for: Overcoming challenges in cartilage regeneration: The role of chondrogenic inducers
Source: Bioeng Transl Med. 2025 Sep 29;11(2):e70079. doi: 10.1002/btm2.70079 (PMC13093458; doi:10.1002/btm2.70079)
Supplement: Supplementary file 1 — Table S1. Chondrogenesis‐inducing compounds by year of discovery. Table S2. Selected cartilage regeneration products. [file BTM2-11-e70079-s001.docx]

Supplementary Table S1: Chondrogenesis‑inducing compounds by year of discovery

| Name | Tested cell lines | Combination tested form | Mechanism | Year | Ref |
| --- | --- | --- | --- | --- | --- |
| Prostaglandin E2 | Embryonic Chick Limb cells | N/A | The cyclic AMP (cAMP) pathway | 1983 | ^1^ |
| Staurosporine | Embryonic limb mesenchyme cells, chick embryo wing bud MSCs | N/A | Mediated through protein kinase C inhibition. through canonical and non-canonical TGF-β pathways | 1991 | ^2^ |
| Dexamethasone | Mouse embryo cells, chicken embryo tibia chondrocytes, murine ATDC5 cells, human chondrosarcoma cells, bovine BMSCs, rabbit-BMSCs. | Polymer scaffold, hydrogel, microsphere | Through enhancing TGFβs-induced phosphorylation of Smads | 1993 | ^3^ |
| TAK-778 | ATDC5 cells | N/A | Via TGF-β | 1999 | ^4^ |
| AG-041R | Mouse bipotent chondroprogenitor cell line, rat articular chondrocytes | N/A | Induction of TGF-β proteins, activates Erk, and p38 MAP kinases | 2003 | ^5^ |
| Simvastatin | Rat intervertebral disc cells, embryonic stem cell (m), rat BMSCs | Nano-complexes | Activated ERK1/2, Wnt/ β-catenin, and p38 kinase pathways | 2008 | ^6^ |
| Melatonin | Human mesenchymal stem cells (human-MSCs), human BMSCs | Injectable hydrogel, polymer scaffold, nanoparticles | Enhanced Smad1 phosphorylation by targeting Smad7, TGF-β) signal pathway | 2009 | ^7^ |

Supplementary Table S1: Chondrogenesis‑inducing compounds by year of discovery (cont*.*)

| Name | Tested cell lines | Combination tested form | Mechanism | Year | Ref |
| --- | --- | --- | --- | --- | --- |
| Kartogenin | Human MSCs, human adipose-derived stem cells (human-ADSCs), Human umbilical cord mesenchymal stem cell (human-UCMSC), human amniotic mesenchymal stromal cells, rat cartilage stem/progenitor cells, rats-ADSCs | Nanoparticles, beads, thermo-gel, nanospheres, fibrin scaffold, injectable hydrogel, nanofibers, polymer scaffold, microsphere | Promoted cartilage matrix synthesis through regulation of the transcriptional program of CBFβ-Runx1 | 2012 | ^8^ |
| Atractylenolide | Rat MSCs | N/A | Via Sonic Hedgehog (Shh) pathway | 2012 | ^9^ |
| Δ5-2-oxopiperazine derivatives | Human BMSCs | N/A | Unrevealed | 2012 | ^10^ |
| TD-198946 | Human BMSCs, human synovium-derived stem cells, pig articular chondrocytes, IPS | Cartilaginous cell-sheet, collagen scaffolds | Regulated Runx1 expression, through the NOTCH3 signalling pathway | 2013 | ^11^ |
| Kaempferol | ATDC5 cells | N/A | Through activation of ERK/BMP-2 signalling pathway | 2013 | ^12^ |
| SB216763 | Human bone marrow stromal cells | N/A | Activated Wnt signalling pathway | 2013 | ^13^ |

Supplementary Table S1: Chondrogenesis‑inducing compounds by year of discovery (cont*.*)

| Name | Tested cell lines | Combination tested form | Mechanism | Year | Ref |
| --- | --- | --- | --- | --- | --- |
| 7,8-dihydroxy-coumarin | Rat adipose-derived MSCs | N/A | Unrevealed | 2013 | ^14^ |
| Resveratrol | Human chondrocyte, human MSCs, horse syndrome-derived MSCs | Microspheres, hydrogel, polymer scaffold. | Via sirtuin-1 (SIRT1) -Sox9 pathway | 2014 | ^15^ |
| Ginsenoside Rg1 | Human ADSCs | N/A | Unrevealed | 2015 | ^16^ |
| H-89 | Human ADSCs | N/A | ERK Pathway Activation | 2016 | ^17^ |
| Honokiol | Human UCMSC | N/A | By blocking nuclear factor-ΚB pathway | 2017 | ^18^ |
| Rosmarinic acid | Rabbit articular chondrocyte | N/A | Activated ERK-1/2 and p38 kinase signalling pathways | 2017 | ^19^ |
| Sesamin | Human amniotic fluid-derived MSCs | N/A | Unrevealed | 2017 | ^20^ |
| 6-bromoindirubin-3′-oxime | Human BMSCs | N/A | Via the Wnt signalling pathway | 2018 | ^21^ |
| 17-DMAG | Human synovial MSCs | N/A | Regulated p21 expression | 2018 | ^22^ |
| Dasatinib | Human Mesenchymal Stem Cells | N/A | Via the Src/Hippo-YAP Signalling Pathway | 2019 | ^23^ |

Supplementary Table S1: Chondrogenesis inducing compounds by year of discovery (cont.)

| Name | Tested cell lines | Combination tested form | Mechanism | Year | Ref |
| --- | --- | --- | --- | --- | --- |
| Fluvastatin | Human adipose derived MSCs, ATDC5 | N/A | Via FGF-3 | 2019 | ^24^ |
| Cycloastragenol | Human- ADMSC | N/A | Unrevealed | 2020 | ^25^ |
| Ellipticine | Human adipose-derived stem cells | N/A | Through p53-based SOX9 regulation | 2020 | ^26^ |
| Rapamycin | Synovium-MSCs | N/A | Through autophagy | 2020 | ^27^ |
| Folic acid | Tonsil-derived mesenchymal stem cells (TMSCs) | N/A | Through CBFβ and RUNX1 | 2022 | ^28^ |
| Pralatrexate | TMSCs | Polypeptide, thermogel | Through the RUNX1-mediated process | 2022 | ^29^ |
| GuiLu-ErXian Glue (GLEXG) | Wharton's jelly-derived MSCs, human MSCs | N/A | Unrevealed | 2023 | ^30^ |
| JD-312 | Mouse bone marrow stem cells | N/A | Mediated via the upregulation of COMP | 2024 | ^31^ |
| Silk sericin | ATDC5 chondrogenic cells | N/A | Via glycolysis and Smad2/3 TGF-β signalling | 2024 | ^32^ |

Supplementary Table S2**:** Selected cartilage regeneration products.

| Name | Components | Indications | Announcement, approval,  or trial phase | Country | Ref |
| --- | --- | --- | --- | --- | --- |
| Bioseed-C | Autologous articular chondrocytes (AAC), Fibrin, polyglycolic acid, polylactic acid and polydioxanone | Focal chondral and osteochondral lesion | 2007  approved | some European countries | ^33^ |
| HyaloFast CE Mark | Hyaluronic acid matrix | Chondral and osteochondral defects of the knee | 2009  phase III | 36 countries | ^34^ |
| Cartistem | Allogeneic umbilical cord blood-derived MSCs, hyaluronic acid hydrogel | Cartilage defects | 2012  approved | South Korea | ^35^ |
| JACC | AAC, Atelocollagen gel | AAC, Atelocollagen gel | Cartilage defects or osteochondritis dissecans | 2012  approved | ^36^ |
| Novocart 3D | AAC, biphasic collagen scaffold | AAC, biphasic collagen scaffold | Articular cartilage defects | 2014  approved | ^37^ |
| MACI | AAC, Porcine type I/III collagen scaffold | Cartilage defects | 2016  approved | USA | ^38^ |
| COPLA cartilage repair device | Collagen and polylactide | Symptomatic full thickness cartilage and osteochondral defects | 2021  preclinical | Estonia, Finland and Sweden | ^39^ |
| CartRevive hydrogel implant | Dextran and hyaluronic acid conjugate | Traumatic cartilage defects | 2023  phase I&II | Netherlands | ^40^ |

**References**

1. Kosher, R. A.; Walker, K. H., The effect of prostaglandins on in vitro limb cartilage differentiation. *Experimental Cell Research* **1983,** 145, (1), 145-153.

2. Kulyk, W. M., Promotion of embryonic limb cartilage differentiation in vitro by staurosporine, a protein kinase C inhibitor. *Dev Biol* **1991,** 146, (1), 38-48.

3. Zimmermann, B.; Cristea, R., Dexamethasone induces chondrogenesis in organoid culture of cell mixtures from mouse embryos. *Anatomy and Embryology* **1993,** 187, (1), 67-73.

4. Akiyama, H.; Fukumoto, A.; Shigeno, C.; Ito, H.; Mukai, S.; Hoshino, T.; Makino, H.; Nakamura, T., TAK-778, a Novel Synthetic 3-Benzothiepin Derivative, Promotes Chondrogenesis in Vitro and in Vivo. *Biochemical and Biophysical Research Communications* **1999,** 261, (1), 131-138.

5. Okazaki, M.; Higuchi, Y.; Kitamura, H., AG-041R stimulates cartilage matrix synthesis without promoting terminal differentiation in rat articular chondrocytes. *Osteoarthritis Cartilage* **2003,** 11, (2), 122-32.

6. Zhang, H.; Lin, C. Y., Simvastatin stimulates chondrogenic phenotype of intervertebral disc cells partially through BMP-2 pathway. *Spine* **2008,** 33, (16), E525-E531.

7. Pei, M.; He, F.; Wei, L.; Rawson, A., Melatonin enhances cartilage matrix synthesis by porcine articular chondrocytes. *J Pineal Res* **2009,** 46, (2), 181-7.

8. Johnson, K.; Zhu, S.; Tremblay, M. S.; Payette, J. N.; Wang, J.; Bouchez, L. C.; Meeusen, S.; Althage, A.; Cho, C. Y.; Wu, X.; Schultz, P. G., A stem cell-based approach to cartilage repair. *Science* **2012,** 336, (6082), 717-21.

9. Li, X.; Wei, G.; Wang, X.; Liu, D.-H.; Deng, R.-D.; Li, H.; Zhou, J.-H.; Li, Y.-W.; Zeng, H.-P.; Chen, D.-F., Targeting of the Sonic Hedgehog Pathway by Atractylenolides Promotes Chondrogenic Differentiation of Mesenchymal Stem Cells. *Biological and Pharmaceutical Bulletin* **2012,** 35, (8), 1328-1335.

10. Cho, T.-J.; Kim, J.; Kwon, S.-K.; Oh, K.; Lee, J.-a.; Lee, D.-S.; Cho, J.; Park, S. B., A potent small-molecule inducer of chondrogenic differentiation of human bone marrow-derived mesenchymal stem cells. *Chem. Sci.* **2012,** 3, 3071-3075.

11. Fumiko, Y.; Hironori, H.; Shinsuke, O.; Atsushi, F.; Yoko, H.; Toshiyuki, I.; Taku, S.; Makoto, H.; Hirotaka, C.; Tsuyoshi, T.; Hiroshi, K.; Ung-il, C., A novel disease-modifying osteoarthritis drug candidate targeting Runx1. *Annals of the Rheumatic Diseases* **2013,** 72, (5), 748.

12. Nepal, M.; Li, L.; Cho, H. K.; Park, J. K.; Soh, Y., Kaempferol induces chondrogenesis in ATDC5 cells through activation of ERK/BMP-2 signaling pathway. *Food and Chemical Toxicology* **2013,** 62, 238-245.

13. Eslaminejad, M. B.; Karimi, N.; Shahhoseini, M., Chondrogenic differentiation of human bone marrow-derived mesenchymal stem cells treated by GSK-3 inhibitors. *Histochemistry and Cell Biology* **2013,** 140, (6), 623-633.

14. Liu, S.; Shao, Y.; Lin, Q.; Liu, H.; Zhang, D., 7,8-Dihydroxy coumarin promotes chondrogenic differentiation of adipose-derived mesenchymal stem cells. *Journal of International Medical Research* **2013,** 41, (1), 82-96.

15. Buhrmann, C.; Busch, F.; Shayan, P.; Shakibaei, M., Sirtuin-1 (SIRT1) is required for promoting chondrogenic differentiation of mesenchymal stem cells. *Journal of Biological Chemistry* **2014,** 289, (32), 22048-22062.

16. Xu, F. T.; Li, H. M.; Zhao, C. Y.; Liang, Z. J.; Huang, M. H.; Li, Q.; Chen, Y. C.; Chi, G. Y., Characterization of Chondrogenic Gene Expression and Cartilage Phenotype Differentiation in Human Breast Adipose-Derived Stem Cells Promoted by Ginsenoside Rg1 in Vitro. *Cellular Physiology and Biochemistry* **2015,** 37, (5), 1890-1902.

17. Choi, E.; Lee, J.; Lee, S.; Song, B.-W.; Seo, H.-H.; Cha, M.-J.; Lim, S.; Lee, C.; Song, S.-W.; Han, G.; Hwang, K.-C., Potential therapeutic application of small molecule with sulfonamide for chondrogenic differentiation and articular cartilage repair. *Bioorganic & Medicinal Chemistry Letters* **2016,** 26, (20), 5098-5102.

18. Wu, H.; Yin, Z.; Wang, L.; Li, F.; Qiu, Y., Honokiol improved chondrogenesis and suppressed inflammation in human umbilical cord derived mesenchymal stem cells via blocking nuclear factor-ΚB pathway. *BMC Cell Biology* **2017,** 18, (1).

19. Eo, S.-H.; Kim, S. J., Rosmarinic acid induces rabbit articular chondrocyte differentiation by decreases matrix metalloproteinase-13 and inflammation by upregulating cyclooxygenase-2 expression. *Journal of Biomedical Science* **2017,** 24, (1), 75.

20. Narakornsak, S.; Aungsuchawan, S.; Pothacharoen, P.; Markmee, R.; Tancharoen, W.; Laowanitwattana, T.; Thaojamnong, C.; Peerapapong, L.; Boonma, N.; Tasuya, W.; Keawdee, J.; Poovachiranon, N., Sesamin encouraging effects on chondrogenic differentiation of human amniotic fluid-derived mesenchymal stem cells. *Acta Histochemica* **2017,** 119, (5), 451-461.

21. Huang, X.; Zhong, L.; Hendriks, J.; Post, J. N.; Karperien, M., The Effects of the WNT-Signaling Modulators BIO and PKF118-310 on the Chondrogenic Differentiation of Human Mesenchymal Stem Cells. *International Journal of Molecular Sciences* **2018,** 19, (2), 561.

22. Bertram, K. L.; Narendran, N.; Tailor, P.; Jablonski, C.; Leonard, C.; Irvine, E.; Hess, R.; Masson, A. O.; Abubacker, S.; Rinker, K.; Biernaskie, J.; Yates, R. M.; Salo, P.; Narendran, A.; Krawetz, R. J., 17-DMAG regulates p21 expression to induce chondrogenesis in vitro and in vivo. *Disease Models & Mechanisms* **2018,** 11, (10).

23. Nie, P.; Li, Y.; Suo, H.; Jiang, N.; Yu, D.; Fang, B., Dasatinib Promotes Chondrogenic Differentiation of Human Mesenchymal Stem Cells via the Src/Hippo-YAP Signaling Pathway. *ACS Biomaterials Science & Engineering* **2019,** 5, (10), 5255-5265.

24. Ishikawa, M.; Ishii, T.; Morikawa, T.; Iijima, Y.; Sueishi, K., The Effects of Fluvastatin on Indian Hedgehog Pathway in Endochondral Ossification. *CARTILAGE* **2021,** 13, (2_suppl), 304S-314S.

25. Szychlinska, M. A.; Calabrese, G.; Ravalli, S.; Parrinello, N. L.; Forte, S.; Castrogiovanni, P.; Pricoco, E.; Imbesi, R.; Castorina, S.; Leonardi, R.; Di Rosa, M.; Musumeci, G., Cycloastragenol as an exogenous enhancer of chondrogenic differentiation of human adipose-derived mesenchymal stem cells. A morphological study. *Cells* **2020,** 9, (2).

26. Lee, J.; Lee, C. Y.; Park, J. H.; Seo, H. H.; Shin, S.; Song, B. W.; Kim, I. K.; Kim, S. W.; Lee, S.; Park, J. C.; Lim, S.; Hwang, K. C., Differentiation of adipose-derived stem cells into functional chondrocytes by a small molecule that induces Sox9. *Exp Mol Med* **2020,** 52, (4), 672-681.

27. Liu, W.; Luo, H.; Wang, R.; Kang, Y.; Liao, W.; Sun, Y.; Chen, G.; Shao, L., Rapamycin-Induced Autophagy Promotes the Chondrogenic Differentiation of Synovium-Derived Mesenchymal Stem Cells in the Temporomandibular Joint in Response to IL-1β. *Biomed Res Int* **2020,** 2020, 4035306.

28. Moon, Y.; Patel, M.; Um, S.; Lee, H. J.; Park, S.; Park, S.-B.; Cha, S.-S.; Jeong, B., Folic acid pretreatment and its sustained delivery for chondrogenic differentiation of MSCs. *Journal of Controlled Release* **2022,** 343, 118-130.

29. Woo, Y.; Patel, M.; Kim, H.; Park, J. K.; Jung, Y.-J.; Cha, S.-S.; Jeong, B., Pralatrexate Sustainably Released from Polypeptide Thermogel Is Effective for Chondrogenic Differentiation of Mesenchymal Stem Cells. *ACS Applied Materials & Interfaces* **2022,** 14, (3), 3773-3783.

30. Yang, Y.-H.; Wen, C.-S.; Kuo, Y.-L.; Fu, S.-L.; Lin, T.-Y.; Chen, C.-M.; Wu, P.-K.; Chen, W.-M.; Wang, J.-Y., GuiLu-ErXian Glue extract promotes mesenchymal stem cells (MSC)-Induced chondrogenesis via exosomes release and delays aging in the MSC senescence process. *Journal of Ethnopharmacology* **2023,** 317, 116784.

31. Gao, J.; Pei, H.; Lv, F.; Niu, X.; You, Y.; He, L.; Hu, S.; Shah, K. M.; Liu, M.; Chen, Y.; Du, B.; Xiong, H.; Luo, J., JD-312 – A novel small molecule that facilitates cartilage repair and alleviates osteoarthritis progression. *Journal of Orthopaedic Translation* **2024,** 44, 60-71.

32. Fongsodsri, K.; Tiyasatkulkovit, W.; Chaisri, U.; Reamtong, O.; Adisakwattana, P.; Supasai, S.; Kanjanapruthipong, T.; Sukphopetch, P.; Aramwit, P.; Ampawong, S., Sericin promotes chondrogenic proliferation and differentiation via glycolysis and Smad2/3 TGF-β signaling inductions and alleviates inflammation in three-dimensional models. *Scientific Reports* **2024,** 14, (1), 11553.

33. Ossendorf, C.; Kaps, C.; Kreuz, P. C.; Burmester, G. R.; Sittinger, M.; Erggelet, C., Treatment of posttraumatic and focal osteoarthritic cartilage defects of the knee with autologous polymer-based three-dimensional chondrocyte grafts: 2-year clinical results. *Arthritis Research & Therapy* **2007,** 9, (2), R41.

34. Anika Therapeutics, I. HyaloFAST Trial for Repair of Articular Cartilage in the Knee (FastTRACK). <https://clinicaltrials.gov/study/NCT02659215>. Accessed 15 Jan 2025.

35. Park, Y. B.; Ha, C. W.; Lee, C. H.; Yoon, Y. C.; Park, Y. G., Cartilage Regeneration in Osteoarthritic Patients by a Composite of Allogeneic Umbilical Cord Blood-Derived Mesenchymal Stem Cells and Hyaluronate Hydrogel: Results from a Clinical Trial for Safety and Proof-of-Concept with 7 Years of Extended Follow-Up. *Stem Cells Transl Med* **2017,** 6, (2), 613-621.

36. Kaibara, T.; Kondo, E.; Matsuoka, M.; Iwasaki, K.; Onodera, T.; Sakamoto, K.; Oda, Y.; Tanei, Z.-i.; Momma, D.; Tanaka, S.; Iwasaki, N., Atelocollagen-associated autologous chondrocyte implantation for the repair of large cartilage defects of the knee: Results at three to seven years. *Journal of Orthopaedic Science* **2024,** 29, (1), 207-216.

37. NIHR NOVOCART 3D for articular cartilage defects of the knee. <https://www.io.nihr.ac.uk/wp-content/uploads/2022/01/13181-Autologous-Chondrocyte-Implant-for-Articular-Cartilage-Defects-V1.0-SEP2019-NON-CONF.pdf>. Accessed 15 Jan 2025

38. ODT Vericel Shares Accelerated Launch Timeline for MACI Arthroscopic Program. <https://www.odtmag.com/breaking-news/vericel-shares-accelerated-launch-timeline-for-maci-arthroscopic-program/>. Accessed 15 Jan 2025.

39. Healthcare, A. Askel Healthcare to Present First Clinical Trial Results of Its COPLA® Knee Cartilage Implant at the 9th Joint Preservation Congress in Warsaw, Poland on June 14-15. <https://www.askelhealthcare.com/askel-healthcare-to-present-first-clinical-trial-results-of-its-copla-knee-cartilage-implant-at-the-9th-joint-preservation-congress-in-warsaw-poland-on-june-14-15-2024/>. Accessed 15 Jan 2025.

40. van der Weiden, G. S.; Mastbergen, S. C.; Both, S. K.; Karperien, M.; Lafeber, F. P.; van Egmond, N.; Custers, R. J. H., Dextran-tryamine hydrogel maintains position and integrity under simulated loading in a human cadaver knee model. *Osteoarthritis and Cartilage Open* **2024,** 6, (3), 100492.
